# DFT and TD-DFT calculations to estimate the photovoltaic parameters of some metal-free dyes based on triphenylamine: the influence of inserting an auxiliary electron-withdrawing group on DSSC's performance†

**DOI:** 10.1039/d5ra04785d

**Published:** 2025-07-28

**Authors:** A. Khadiri, Abhinay Thakur, I. Warad, H. Zarrok, H. Oudda, M. Beraich, L. Bazzi, A. Zarrouk

**Affiliations:** a Laboratory of Advanced Materials and Process Engineering, Faculty of Sciences, Ibn Tofail University P.O. Box. 133 Kenitra 14000 Morocco; b Division of Research and Development, Lovely Professional University Phagwara Punjab 144411 India; c Department of Chemistry, AN-Najah National University P.O. Box 7 Nablus Palestine; d Laboratory of Applied Sciences for Sustainable Development, Higher School of Technology of El Kelaa Des Sraghna, Cadi Ayyad University Marrakech Morocco; e Laboratory of Materials, Energy and Environment, Faculty of Sciences Semlalia, Cadi Ayyad University Marrakech Morocco; f Laboratory of Industrial Engineering, Energy and Environment (LI3E) SupMTI Rabat Morocco azarrouk@gmail.com +212-00212665201397; g Laboratory of Materials, Nanotechnology and Environment, Faculty of Sciences, Mohammed V University in Rabat Av. Ibn Battouta, PO Box 1014, Agdal-Rabat Morocco

## Abstract

Using density functional theory (DFT) and time-dependent density functional theory (TD-DFT), the power conversion efficiency (PCE) of a series of novel designed D-π-Aa-A metal-free dyes with different auxiliary electron-withdrawing groups is predicted and discussed. The effect of incorporating an auxiliary electron-withdrawing group (Ap) between the π-bridge and anchoring group (A) on the photovoltaic properties is investigated and discussed. The key optoelectronic parameters that can be used for estimating PCE of the investigated dyes in DSSCs like FMOs and gap energies, light harvesting efficiency (LHE), the energy of the conduction band of the adsorbed dye cluster, electron, hole, and total reorganization energies, driving energy of electron injection and regeneration, natural bond orbital charge (NBO), dipole moment, and the chemical reactive parameters are determined and discussed. The predicted PCE of 7.15% shows a good agreement with the experimental results of 7.3%, confirming our methodology's credibility and validity. In addition, the predicted PCEs of our designed organic compounds are 8.52, 9.50, 10.77, 11.62, and 12.45%, indicating that the present work can provide new clues for synthesizing efficient organic compounds for DSSCs.

## Abstract 2

Dyes confirmed accurate photovoltaic performance and charge transfer behavior.

## Graphical abstract

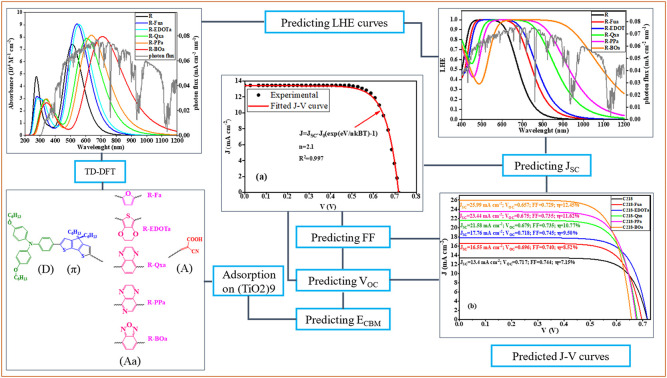

## 1. Introduction

Energy is one of the major inputs for all countries' economic and societal development. The most used energy sources in the world are fossil fuels such as petroleum, coal, natural gas, and oil. These sources are being depleted, pollutants for the environment, and cause real climate change. However, energies obtained from renewable sources such as biomass, wind, wave, hydropower, geothermal, solar, and so on can solve the energy crisis and environmental pollution.^1^ Among all the energy sources cited above, solar energy stands out as a potential alternative energy thanks to its availability, being clean and green, sustainable, affordable, and environmentally friendly.^2^ Therefore, Fuller, Chapin, and Pearson invented the 1st generation solar cells based on silicon to convert solar radiation into electrical power.^3^ These cells have many advantages, such as a high-power conversion efficiency of 26% and a lifespan of more than 20 years. Still, they have several disadvantages, such as high cost, low efficiency in low light conditions, environmental impact of manufacturing, and so on. The 2nd generation solar cells, which are characterized by lightweight design and good flexibility, are based on thin-film technologies using silicon materials like polycrystalline Si, microcrystalline Si, micromorph tandem Si, and amorphous Si, or other materials such as gallium arsenide, cadmium telluride, copper indium selenide, and so on.^4^ These cells suffer from several drawbacks, such as being easily degradable, highly toxic, rare, and expensive.^5^ Therefore, the 3rd generation, classified as non-silicon-based cells such as perovskite solar cells (PSCs), organic solar cells (OSCs), and especially dye-sensitized solar cells (DSSCs), has been invented and developed to reduce the cost and overcome the disadvantages of the first and second generations. Since their invention by O'Regan and Gratzel,^6^ DSSCs have significantly attracted more attention thanks to their flexibility, simple fabrication process, design attractiveness, environmentally friendly, adjustable optical properties, low fabrication costs, and considerable PCE. In general, DSSCs contain several components like a metal–semiconductor (TiO_2_), a counter electrode, a redox electrolyte that contains the (I^−^_3_/I^−^) couple, and especially the photosensitizers (dye molecules). When the sunlight hits the electrons in the dyes, they absorb photons and become excited; the excited dyes release photoelectrons and inject them into the TiO_2_. Then, the electrons moving in the external circuit are collected by the counter electrode (CE). After that, the oxidized dyes will be regenerated by the redox electrolyte. The counter electrode (CE) plays a vital role in dye-sensitized solar cells (DSSCs), particularly in catalyzing the reduction of the redox couple and facilitating charge transport. Numerous studies have focused on improving counter electrode materials to enhance DSSC performance and reduce overall costs. Various types of CEs have been investigated,^7,8^ including metal-based electrodes such as platinum^9,10^ and platinum-nickel alloys;^11,12^ carbon-based materials such as carbon black,^13^ carbon nanotubes,^13^ carbon nanofibers,^14^ graphene,^15^ and mesoporous carbon;^16^ as well as conductive polymers like poly(3,4-ethylenedioxythiophene) (PEDOT),^16^ polypyrrole (PPy),^17^ and polyaniline (PANI).^18^ On the other hand, the sensitizer plays the principal role in DSSCs for converting sunlight into electricity. Therefore, many researchers and scientists have designed, synthesized, investigated, and studied different organic and organometallic compounds as sensitizers for ameliorating DSSCs' performance. Among all organic-based dyes used up to now as sensitizers in DSSCs, we cite carbazole, phenothiazine, perylene, and triphenylamine dyes.^2,19–24^ However, zinc porphyrins and ruthenium complexes are selected as the efficient organometallic dyes tested in DSSCs.^2,25^ Using experimental studies to evaluate an organic compound as an efficient dye sensitizer in DSSCs leads, in most cases, to low efficiency, requiring a long time and more expensive. Therefore, using the theoretical calculations to confirm the experimental results and estimate the photovoltaic parameters before the experimental studies can help authors obtain more information about the investigated organic compounds and give new clues for experimentation to discover and select new efficient dyes for DSSCs. For this purpose, many scientists have theoretically studied many metal-free organic compounds as sensitizers in DSSCs. Caibin Zhao *et al.*^26^ studied theoretically using DFT and TD-DFT calculations eight novel designed organic compounds as sensitizer dyes in DSSCs by modifying different internal acceptors in the ZL003 reference dye. The predicted short circuit current density *J*_SC_, open circuit photovoltage *V*_OC_, fill factor FF, and PCE for ZL003 reference dye take, respectively, 20.21 mA cm^−2^, 0.966 V, 0.688, and 13.42%, indicating good agreement with the experimental results of 20.73 mA cm^−2^, 0.956 V, 0.685, and 13.6%. In addition, the results show that the PCE enhanced and took 15.38, 15.80, and 19.93% when the benzothiadiazole (BT) in ZL003 reference dye is replaced by benzoselenadiazole (dye M6), selenadiazolopyridine (dye M7), and thienopyrazine (dye M4), respectively. Based on the principal energies of DSSCs, Z. Yang *et al.*^27^ have introduced *K*_*J*_SC__, *K*_*V*_OC__, and *K*_*P*_max__ as the correction parameters of *J*_SC_, *V*_OC_, and FF, respectively, to minimize the absolute fractional errors between the experimental and the theoretical results. By employing DFT and TD-DFT methods for six reference organic compounds abbreviated as L0, L1, L2, C281, WS-92, and WS-2, the results showed that the maximum fractional errors are less than 17.3% for *J*_SC_, less than 11.4% for *V*_OC_, and less than 4.22% for PCE, indicating the validity of their proposed model. Based on JY56 reference dye,^28^ L. Mao *et al.*^29^ studied the influence of introducing various π-bridges on the photovoltaic performances for DSSC by employing DFT and TD-DFT methods. The predicted values of 684 mV, 14.84 mA cm^−2^, 0.84, and 8.55% of *V*_OC_, *J*_SC_, FF, and PCE, respectively, agree well with the experimental values of 816 mV, 16.41 mA cm^−2^, 0.61, and 8.19% for JY56 reference dye. The results show that when they substitute the original π-spacer (DTP) in JY56 reference dye with other π-spacers abbreviated by CDT, RD, and NDI, the predicted PCE takes 9.73, 7.11, and 15.51%, respectively, indicating that the designed dyes with CDT and NDI groups as π-spacers can enhance the photovoltaic performances for DSSCs. Using DFT and TD-DFT calculations, A. U. Rahman *et al.*^30^ designed and predicted the photovoltaic parameters for five BODIPY-carbazole (D-π-A-A) dyads. Firstly, they added, on the D0-based dye, the dimethylamine (–N(CH_3_)_2_) as an electron-donating unit at the electron-rich carbazole group and the carboxyl unit (–COOH) as an electron-accepting moiety at the BODIPY core (dye D1). Then, they incorporated between the BODIPAY core and carboxyl groups different auxiliary acceptors such as thiophene (dye D1T), furan (dye D1F), and phosphole (dye D1P). The results show that the D1P dye is selected as the efficient dye in the series with a PCE of 13.5%, followed by D1F (12.13%), D1T (11.25%), and D1 (8.93%). Based on the DH-16 reference dye,^31^ Panpan Heng *et al.*^32^ studied a novel-designed series of triphenylamine-based dyes. Firstly, they simplified the chemical structure of the reference dye by replacing the alkyl groups with ethyl groups (dye 1). The dye 2 is obtained by altering the benzothiadiazole electron-withdrawing group (BTZ) and carbazole-based π-spacer (CZ-based π-spacer) in dye 1. The dye 3 is obtained by replacing the BTZ with another electron-withdrawing group, namely 2-ethyl-2*H*-naphtho(2,3-*d*)(1,2,3)triazole (ENT). The dye 4 is obtained by replacing the carbazole-based π-spacer with the thieno(3,2-*b*)thiophene (TT) group. For the DH-16 reference dye, the theoretical calculations show that the predicted 833 mV, 10.31 mA cm^−2^, 0.86, and 7.42% of *V*_OC_, *J*_SC_, FF, and PCE agree very well with the experimental results of 900 mV, 10.55 mA cm^−2^, 0.71, and 6.76%, confirming the validity of the proposed estimating model. For the novel-designed dyes, the predicted PCE takes 12.94%, 8.60%, and 16.49% for dye 2, dye 3, and dye 4, respectively, indicating that this work can help authors to synthesize efficient organic compounds for improving the photoelectronic performance in DSSCs.

**Fig. 1:**
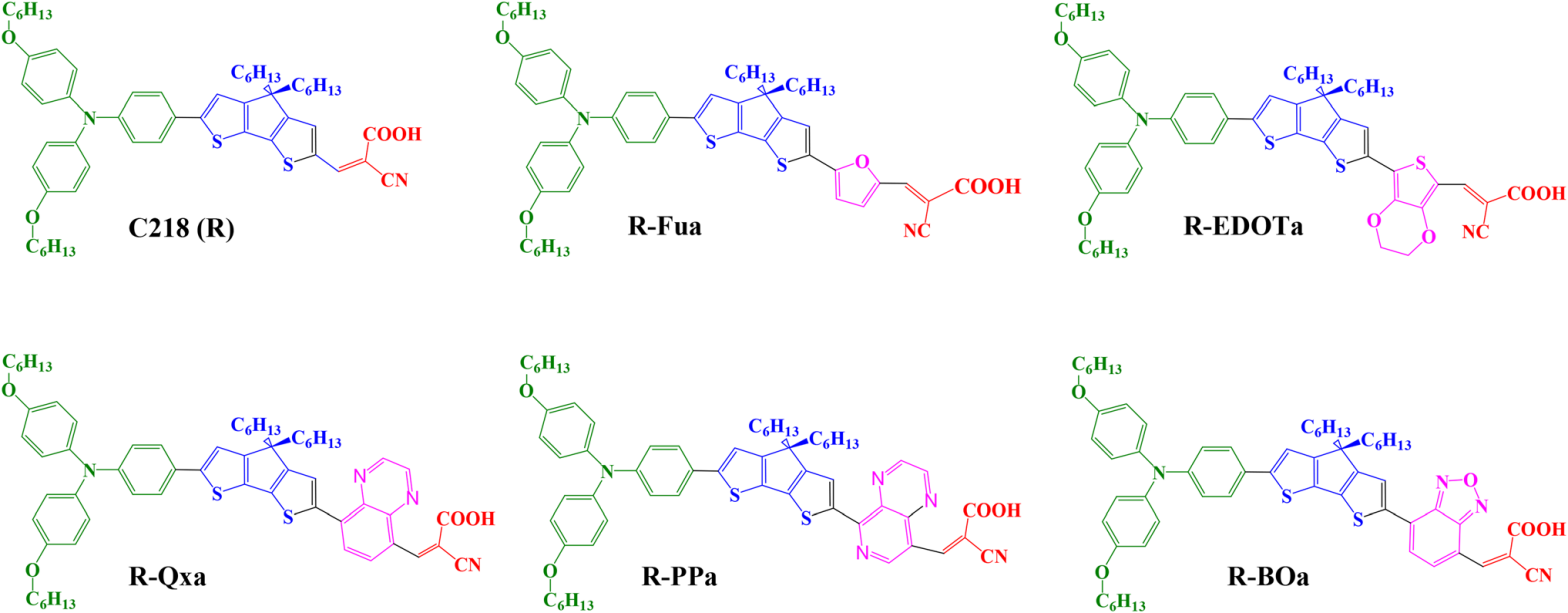
Chemical structure of molecular organic compounds studied in this work.

In this work, to find the efficient organic compounds that can be used as dye sensitizers in DSSCs, we have studied and investigated theoretically a novel-designed series of triphenylamine-based dyes by introducing different auxiliary electron-withdrawing groups (Ap) in C218 as a reference dye (R).^33,34^ The auxiliary electron-withdrawing groups used in this study which are commonly used to enhance the PCE of DSSCs are 2,1,3-benzooxadiazole (BO),^35–37^ pyrido(3,4-*b*) pyrazine (PP),^38–40^ quinoxaline (Qx),^41–44^ furan (Fu),^45,46^ and 3,4-Ethylenedioxythiophene (EDOT)^47–50^ (show Fig. 1). In order to study the influence of incorporating the Ap groups on the photovoltaic properties, the both DFT and TD-DFT calculations are considered to extract the optoelectronic parameters for each free dye and adsorbed dye@TiO_2_ clusters. Then, we constructed a theoretical strategy to estimate the photovoltaic factors like *V*_OC_, *J*_SC_, FF, and PCE. The predicted 0.717 V of *V*_OC_, 13.4 mA cm^−2^ of *J*_SC_, 74.4% of FF, and 7.15% of PCE agree well with the experimental 0.721 of *V*_OC_, 13.5 mA cm^−2^ of *J*_SC_, 75.3% of FF, and 7.3% of PCE,^34^ confirming that our strategy for estimating the photovoltaic parameters is valid and credible.

## 2. Computational methods

In this work, all DFT and TD-DFT calculations were performed using the Gaussian 09 software.^51^ To choose the suitable function for optimizing the molecular geometry of the reference dye and simulating its UV-vis absorption spectrum, we have used different functions such as HSEH1PBE,^52^ WB97XD,^53^ B3LYP,^54^ CAM-B3LYP,^55^ and MPW1PW9 (ref. 56) in conjunction with different 6-31G, 6-31G(d), 6-31G(d, p), 6-311G, 6-311G(d), and 6-311G** basis sets under CPCM in CH_2_Cl_2_ (DCM) solvent, the results are illustrated in Tables SD1 and SD2 in ESI data.† By comparing the experimental and the theoretical results, B3LYP/6-311G** is selected as a suitable functional for optimizing the geometry, and CAM-B3LYP/6-311G** functional is considered for simulating the UV-vis absorption spectrum of the reference dye. Therefore, these two functionals were used to optimize the neutral and charged dyes and simulate the UV-vis absorption spectra of all investigated dyes. To study the adsorption properties of the designed organic compounds on the metal–semiconductor surface, we have chosen (TiO_2_)_9_ as a model. This (TiO_2_)_9_ cluster, as well as the adsorbed dye@(TiO_2_)_9_ systems, are also optimized using B3LYP functions with two basis sets, LanL2DZ for the Titanium atoms and 6-311G** for the H, C, N, O, and S atoms. However, the CAM-B3LYP function in conjunction with the above basis set is considered to simulate their UV-vis absorption spectra.^57,58^ Finally, we have used the Multiwfn 3.8 software^59,60^ to extract more information about each dye's properties such as the percentage contribution of each moiety (D, π, Ap, and A) in HOMO, LUMO, hole, and electron, the intra-fragment electron redistribution, the HOMO and LUMO contributions in holes and electrons, the quantity of the transferred intramolecular electron, total TDOS, and PDOS, and so on.

## 3. Results and discussion

### 3.1. Optimized geometries of the free dye molecules

**Fig. 2:**
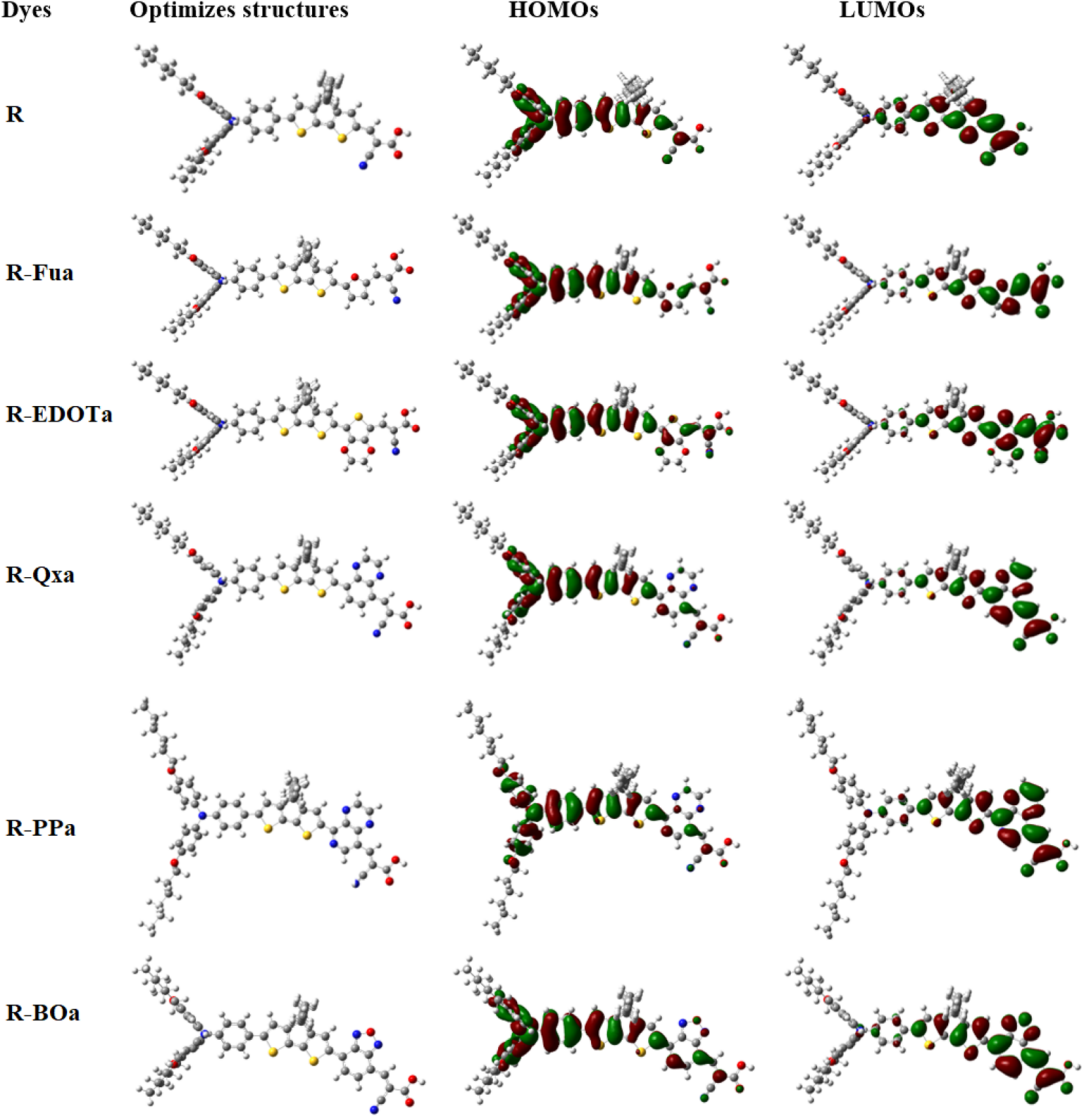
The HOMOs, LUMOs, and optimized structures of the investigated dyes in DCM medium with 0.02 au as isovalue.

**Scheme 1:**
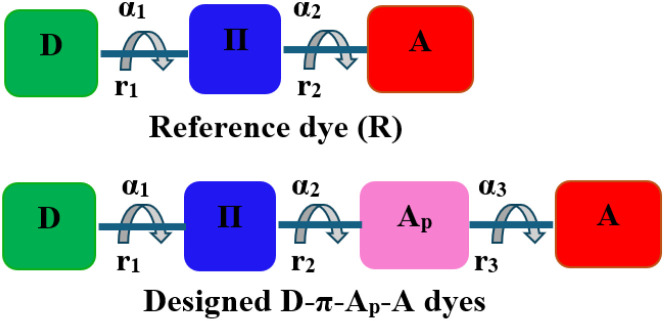
Schematic structures for reference (R) and designed organic compounds.

The optimized molecular geometries of the designed and reference organic compounds are illustrated in Fig. 2. The bond lengths (*r*_1_, *r*_2_, and *r*_3_), dihedral angles (*α*_1_, *α*_2_, and *α*_3_) as well as their absolute deviation (Δ*α*_1_, Δ*α*_2_, and Δ*α*_3_) from the plane dihedral angles (180° or −180°) between different groups as represented in Scheme 1 are tabulated in Table 1. The results show that the values of Δ*α*_1_ are between 18.33° and 25.75° in the vacuum level and between 17.9° and 24.65° in DCM medium, which enhances thermal stability and decreases charge aggregation.^61^ Except for the small Δ*α*_2_ and Δ*α*_3_ of R-EDOTa, all other dyes show a coplanar dihedral angle between the π, Ap, and A groups, which exhibits excellent conjugation properties between them. In addition, the *r*_1_, *r*_2_, and *r*_3_ bond lengths are between 1.41 and 1.46 Å, these values are above the C=C double bonds (1.43 Å) and under the C–C single bonds (1.54 Å),^62^ indicating that the investigated organic compounds present a strong resonance structure that can improve the charge transfer from the D group to the A group throughout the π and Ap groups.

**Table 1:** The calculated bond distances *r*_1_, *r*_2_, and *r*_3_ (in Å), the dihedral angles *α*_1_, *α*_2_, and *α*_3_ (in °), and the absolute deviation Δ*α*_1_, Δ*α*_2_, and Δ*α*_3_ from the plane dihedral angles of the studied dyes in DCM medium and in vacuum level

Dyes	Levels	*r*_1_ (Å)	*r*_2_ (Å)	*r*_3_ (Å)	*α*_1_ (°)	Δ*α*_1_ (°)	*α*_2_ (°)	Δ*α*_2_ (°)	*α*_3_ (°)	Δ*α*_3_ (°)
R	Vacuum	1.459	1.416	—	154.77	25.23	−179.86	0.14	—	
R-Fua		1.460	1.428	1.412	154.25	25.75	179.46	0.54	−179.87	0.13
R-EDOTa		1.460	1.431	1.418	154.26	25.74	176.79	3.21	−160.74	19.26
R-Qxa		1.458	1.446	1.442	161.67	18.33	−179.05	0.95	−179.90	0.10
R-PPa		1.457	1.433	1.440	−157.30	22.70	179.42	0.58	179.33	0.67
R-BOa		1.457	1.431	1.437	156.48	23.52	179.62	0.38	179.95	0.05
R	DCM	1.458	1.408	—	155.75	24.25	−179.89	0.11	—	—
R-Fua		1.460	1.425	1.405	155.35	24.65	179.34	0.66	−179.81	0.19
R-EDOTa		1.460	1.428	1.409	155.38	24.62	176.65	3.35	−164.98	15.02
R-Qxa		1.458	1.443	1.435	162.10	17.90	−179.04	0.96	−179.31	0.69
R-PPa		1.456	1.428	1.433	−159.74	20.26	179.16	0.84	179.22	0.78
R-BOa		1.456	1.425	1.429	157.83	22.17	179.84	0.16	−179.80	0.20

### 3.2. FMOs, PDOS, and NBOs

**Fig. 3:**
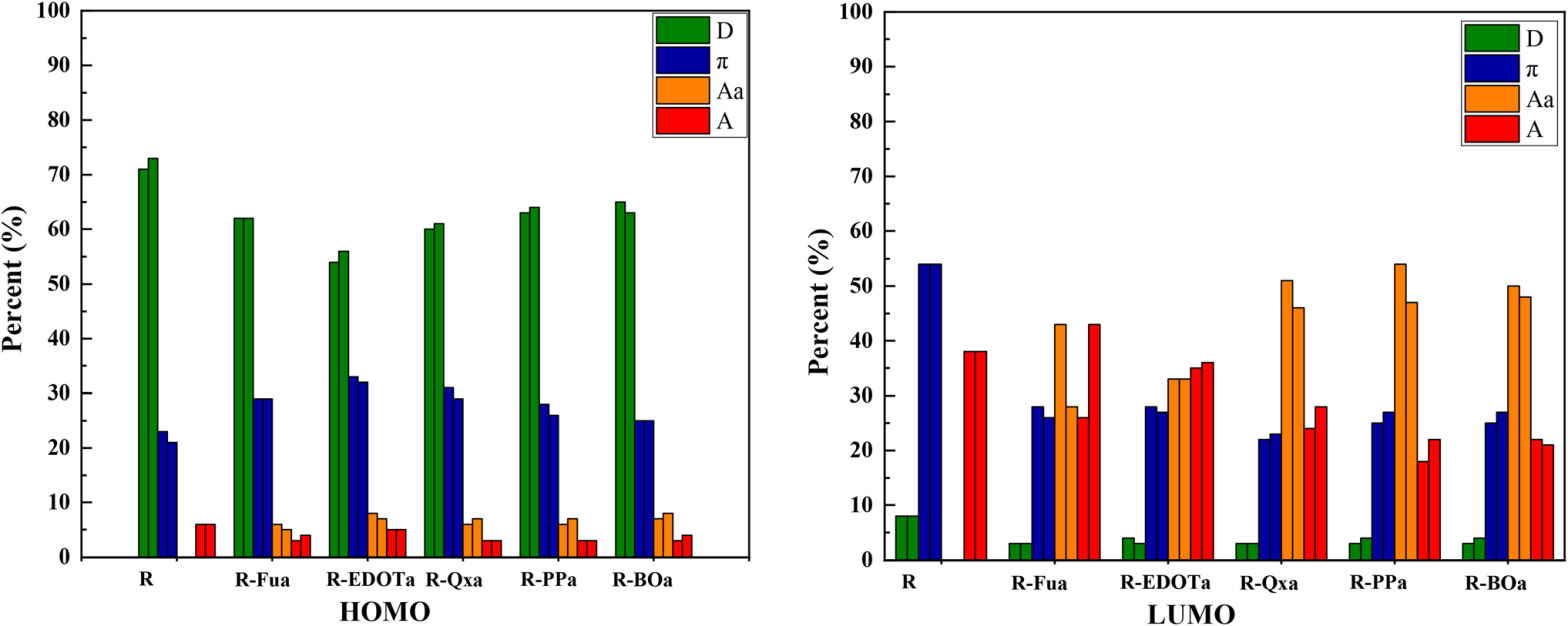
Contributions (in %) in FMOs by each group in different dyes in DCM medium (right) and vacuum level (left).

The distribution of the FMOs of the investigated organic compounds is illustrated in Fig. 2. The HOMOs for all organic compounds are primarily distributed on the D and π-spacer. The LUMO orbital for the R compound is primarily distributed on the π-A groups. However, for the designed D-π-Ap-A organic compounds, the LUMOs are primarily distributed on the π-Ap-and A groups. To confirm these observations as well as extract the percentage contribution of the above groups in HOMO and LUMO and determine their donating or accepting characters, PDOS analysis was used to calculate the corresponding group contributions using the Multiwfn 3.8 program.^59,60^ The percentage contribution in FMOs by different groups of molecules reflects their nature, if the contribution of a group is more visible in the LUMO, it can function as an electron-withdrawing group. In contrast, it can function as an electron donor group if its contribution is more visible in the HOMO.^63^ As illustrated in Table 2 and Fig. 3, the percentage contribution in the HOMO by the triphenylamine group (D) ranges from 54 to 73% and its contribution in the LUMO ranges from 3 to 8%, confirming its electron-donating character. In contrast, the CAA group contributes more in the LUMO (ranging from 18 to 41% in the vacuum level and from 21 to 43% in the DCM medium) than its contribution in the HOMO (ranging from 3 to 6% in both vacuum and solvent environment), confirming the electron-withdrawing character of the corresponding group. The contribution of the Ap groups is more pronounced in the LUMO (ranging from 33 to 48%) than their low contributions in the HOMO (ranging from 5 to 8%), confirming their electron-withdrawing character. Indeed, the percentage contribution of these groups increases in the order Fua < EDOTa < Qxa < PPa < BOa in both vacuum and solvent media, indicating that the BO groups present a more accepting ability among all Ap tested. The π-spacer group contribution in the HOMO (21%) is less than its contribution in the LUMO (54%) for the reference dye and its contribution in both HOMO and LUMO orbitals is nearly identical for the designed dyes, indicating that this unit can act as a redistributor group by accepting electrons from the D group and transferring them to the Ap and A groups. Indeed, the results show that when we introduce the Fu, EDOT, Qx, PP, and BO groups as an Ap groups, the percentage contribution of the D unit in both HOMO and LUMO orbitals decreases, increases slightly in the HOMO and decreases about half in the LUMO of the π-bridge, indicating that the introduction of the above groups between the π-spacer and A groups can facilitate the intramolecular charge transfer (ICT) and consequently enhance their efficiency in DSSCs.

**Table 2:** NBO charges (in e) of each group and their percentage contributions (%) to the FMOs in DCM medium and vacuum level

Dyes	Levels	HOMO				LUMO				NBO (e)			
		D	π	Ap	A	D	π	Ap	A	D	π	Ap	A
R	Vacuum	71	23	—	6	8	54	—	38	0.048	0.166	—	−0.214
R-Fua		62	29	5	4	4	28	27	41	0.033	0.079	0.094	−0.207
R-EDOTa		54	33	8	5	4	28	33	35	0.024	0.077	0.100	−0.201
R-Qxa		60	31	6	3	3	22	51	24	0.039	0.150	−0.073	−0.116
R-PPa		63	28	6	3	3	25	54	18	0.051	0.209	−0.142	−0.118
R-BOa		65	25	7	3	3	25	50	22	0.057	0.192	−0.128	−0.120
R	DCM	73	21	—	6	8	54	—	38	0.062	0.220	—	−0.282
R-Fua		62	29	5	4	3	26	28	43	0.036	0.116	0.114	−0.266
R-EDOTa		56	32	7	5	3	27	33	36	0.029	0.106	0.142	−0.276
R-Qxa		61	29	7	3	3	23	46	28	0.048	0.179	−0.057	−0.171
R-PPa		64	26	7	3	4	27	47	22	0.068	0.238	−0.137	−0.169
R-BOa		63	25	8	4	4	27	48	21	0.076	0.255	−0.147	−0.184

Calculating NBO charge can be used for estimating the electron-pushing or withdrawing ability of different parts of dye molecules and understanding the charge distribution and the ICT mechanism in the corresponding parts.^64–66^ The calculated NBO charges of each unit in the investigated organic compounds are tabulated in Table 2. The NBO charges of the D and π-spacer groups are positive for all studied dyes, confirming the electron-donating characters of these groups. In contrast, the NBO charges of the A and Ap groups (except Fu and EDOT) are negative, confirming their electron-withdrawing characters. Consequently, the photoelectrons can be transferred from the D and π-spacer groups to the A group throughout the auxiliary electron-withdrawing group (Ap). We can note that the calculated NBO charges in DCM medium for the D, π-spacer, Fu, and EDOT groups are more positive than their values at the vacuum level. However, for the A, Qx, PP, and BO groups, the NBO charges are more negative in DCM medium, indicating that the solution effect ameliorates the charge-separated state for dyes.

### 3.3. *E*_HOMO_, *E*_LUMO_, *E*_gap_, and dipole moment of the free organic compounds

**Fig. 4:**
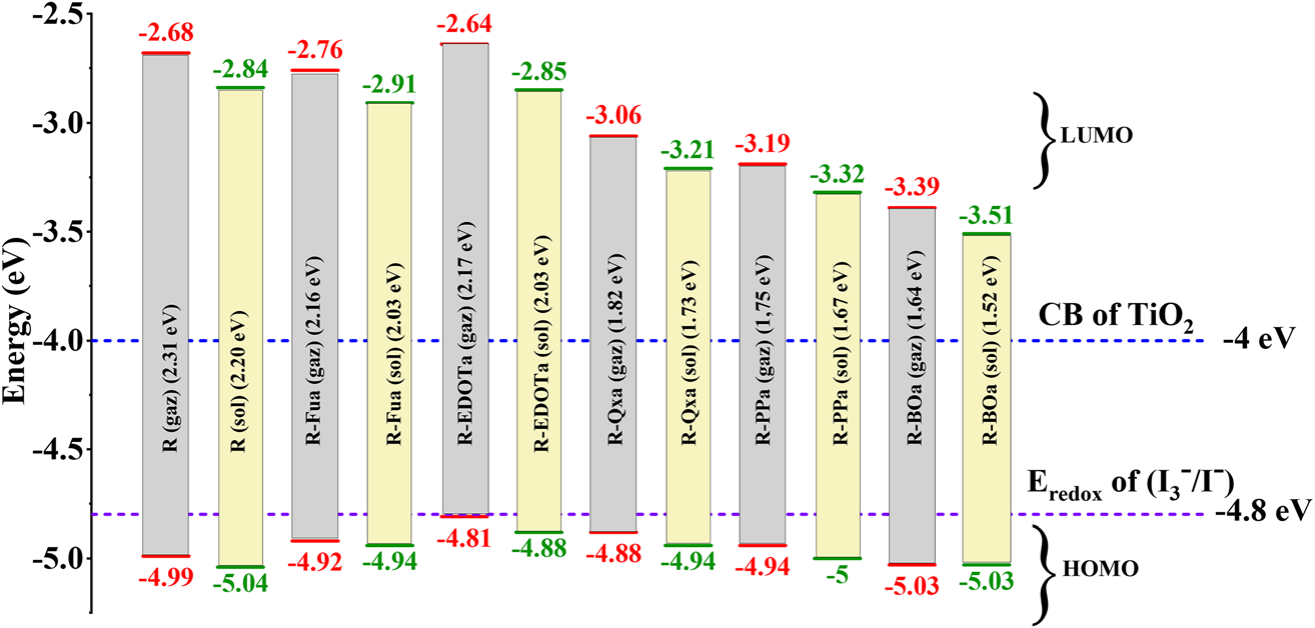
The schematic energy diagram of each organic compound in DCM medium and vacuum level.

To study the spontaneity effect of the electron injection from the oxidized dyes to the CB of the TiO_2_ and the dyes regeneration by the electrolyte, LUMO, HOMO, and gap energies were calculated and illustrated in Table 3 and Fig. 4. The results show that the LUMO energy of all dyes is located above the TiO_2_ semiconductor CB (−4.0 eV), confirming the spontaneity of the electron injection from the dyes to the semiconductor.^67^ In addition, the HOMO energy of all dyes is positioned below the redox potential (−4.8 eV) of the (I^−^_3_/I^−^) couple, indicating that the oxidized dyes can be regenerated spontaneously by the reducing ion (I^−^).^68^ Furthermore, gap energy is a crucial factor that can provide more information about dye efficiency when this dye is used as a sensitizer in DSSC. The calculated gap energies are all lower than the R dye's gap and increase in the order R > R-Fua = R-EDOTa > R-Qxa > R-PPa > R-BOa in both vacuum and solvent levels, indicating that the insertion of an Ap group decreases the gap energy of the designed organic compounds which leads to facilitate the electron transition between FMOs, broaden the UV-vis absorption spectra, redshift the maximum absorption wavelength (*λ*_max_), improve ICT, and consequently enhance the PCE of DSSC.^69^ Indeed, all HOMO and LUMO energies in the DCM medium are positioned below their values in the vacuum level and the gap energies are all small in the solvent than in the vacuum, indicating that the optoelectronic properties can be improved by using the DCM medium.

**Table 3:** The calculated LUMO, HOMO, and gap energies (in eV) of each organic compound in the vacuum level and DCM medium

Dyes	Media	*E*_LUMO_ (eV)	*E*_HOMO_ (eV)	Gap (eV)	*μ* (D)
R	Vacuum	−2.68	−4.99	2.31	13.35
R-Fu-a		−2.76	−4.92	2.16	15.29
R-EDOT-a		−2.64	−4.81	2.17	12.81
R-Qx-a		−3.06	−4.88	1.82	14.73
R-PP-a		−3.19	−4.94	1.75	13.36
R-BO-a		−3.39	−5.03	1.64	14.21
R	DCM	−2.84	−5.04	2.20	16.94
R-Fu-a		−2.91	−4.94	2.03	19.10
R-EDOT-a		−2.85	−4.88	2.03	17.06
R-Qx-a		−3.21	−4.94	1.73	18.46
R-PP-a		−3.32	−5.00	1.67	18.15
R-BO-a		−3.51	−5.03	1.52	19.47

The dipole moment is one of the key parameters that can affect the efficiency of the dyes in DSSCs; their corresponding values are tabulated in Table 3. The corresponding values increase when we introduce Fu, EDOT, Qx, PP, and BO as auxiliary electron-withdrawing groups, indicating that the designed dyes will be more efficient than the reference dye.^70^ Indeed, the solvation effect with DCM enhances the dipole moment more than in the vacuum medium, confirming the enhancement of the charge-separated state for dyes.

### 3.4. Ultraviolet-visible absorption and emission spectra, excited state lifetime, electronic injection, and dye regeneration driving forces

**Fig. 5:**
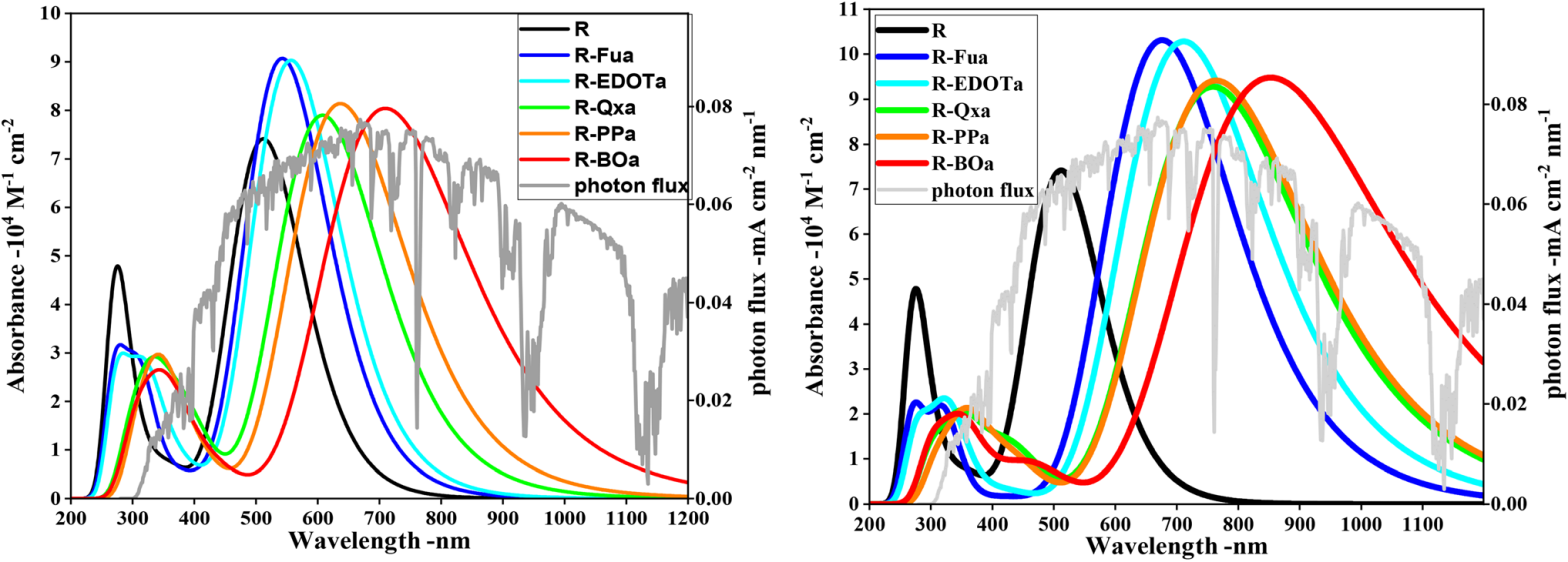
Absorption spectra (on left) and emission spectra (on right) of the organic compounds performed by employing TD-DFT/CAM-B3LYP/6-311G** under CPCM in DCM medium.

To function as an effective sensitizer, the dye upon photoexcitation should harvest more sunlight in the visible (Vis) and near-infrared (NIF) domains. By employing TD-DFT/CAM-B3LYP/6-311G** in DCM medium under CPCM, the absorption spectra of the ten lowest singlet–singlet excitations of the free compounds were calculated and illustrated in Fig. 5. The corresponding parameters like the oscillator strengths (*f*), the absorption maximum wavelengths (*λ*_max_), the vertical excitation energies (*E*_ex_), and the main compositions are illustrated in Table 4 and SD3. The UV-vis absorption spectra for all free dyes are similar and show two bands, one small and one large. The small band that appears in the UV domain (between 200 and 400 nm) can be assigned to n → π* and/or π → π* transitions. The large band that appears in the Vis and NIR domains can be ascribed to the ICT between different groups in D → π → Ap → A direction and/or π → π* transition. The insertion of Fu, EDOT, Qx, PP, and BO as auxiliary electron-withdrawing groups reduces the excitation energy *E*_ex_, enhances the oscillator strength (*f*), and shifts the maximum absorption wavelength (*λ*_max_) to the red and NIR domains. The excitation energy of the first excited state (S1) decreases in the order: R (2.42 eV) > R-Fua (2.28 eV) > R-EDOTa (2.23 eV) > R-Qxa (2.04 eV) > R-PPa (1.95 eV) > R-BOa (1.75 eV). The oscillator strength *f* increases the order R (1.83) < R-Qxa (1.95) < R-BOa (1.99) < R-PPa (1.95) < R-EDOT (2.23) < R-Fu (2.24). The maximum absorption wavelength *λ*_max_ increases in the order: R (512 nm) < R-Fua (543 nm) < R-EDOTa (557 nm) < R-Qxa (608 nm) < R-PPa (637 nm) < R-BOa (710 nm), this trend show that inserting Fu, EDOT, Qx, PP, and BO as auxiliary electron-withdrawing groups leads to a red shifting of *λ*_max_ by 33 nm, 45 nm, 96 nm, 125 nm and 198 nm, respectively, indicating a good agreement with the gap energy of the free dyes, *i.e.*, the more the gap energy is small the more the absorption spectrum is large and shift to red and infrared domains. In addition, the excitation is dominated by the HOMO → LUMO transition (between 61 and 67%) followed by the HOMO-1 → LUMO transition (between 24 and 29%), indicating that the CT dominates the intramolecular transition.

**Table 4:** Absorption parameters, driving force of electron injection (Δ*G*_inj_), and dye regeneration Δ*G*_reg_ of the free dyes in DCM medium (all energies in eV)

Dyes	State	*λ*_max_ (nm)	FWHM (nm)	*E*_ex_ (eV)	*f*	Main compositions (%)	*E*_dye_ (eV)	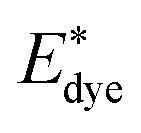 (eV)	Δ*G*_inj_ (eV)	Δ*G*_reg_ (eV)	*S*
R	S_1_	512	118	2.42	1.83	H → L (65); H−1 → L (29)	5.04	2.62	−1.38	−0.24	1
R-Fua	S_1_	543	162	2.28	2.24	H → L (61); H−1 → L (27)	4.94	2.66	−1.34	−0.14	1.30
R-EDOTa	S_1_	557	171	2.23	2.23	H → L (67); H−1 → L (24)	4.88	2.65	−1.35	−0.08	1.37
R-Qxa	S_1_	608	205	2.04	1.95	H → L (67); H−1 → L (24)	4.94	2.90	−1.10	−0.14	1.46
R-PPa	S_1_	637	225	1.95	2.01	H → L (68); H−1 → L (24)	5.00	3.05	−0.95	−0.20	1.59
R-BOa	S_1_	710	281	1.75	1.99	H → L (67); H−1 → L (26)	5.03	3.28	−0.72	−0.23	1.92

To compare the light-absorbing power of the studied dyes, we have introduced a new parameter (*S*) called the normalized integrated spectral curve area,^58,71^ which is calculated by dividing the spectral area of each dye by that of the reference dye. The results in Table 4 show that this parameter for all designed dyes takes large values than the R dye and increases in the order: R (1) < R-Fua (1.30) < R-EDOTa (1.37) < R-Qxa (1.46) < R-PPa (1.59) < R-BOa (1.92), confirming that our designed dyes can absorb more sunlight, ameliorate the short circuit current density *J*_SC_, and improve the PCE. On the other hand, the FWHM (Full Width at Half Maximum) parameter plays an important role in light-harvesting efficiency (LHE). A larger FWHM value indicates a broader absorption band, which can enhance the dye's ability to harvest sunlight. The FWHM values determined at *λ*_max_ increase in the following order: R (118 nm) < R-Fua (162 nm) < R-EDOT (171 nm) < R-Qxa (205 nm) < R-PPa (225 nm) < R-BOa (281 nm), suggesting that the sunlight absorption capability improves along this sequence. Consequently, the designed dyes are expected to be more efficient than the reference dye in terms of light harvesting.

**Fig. 6:**
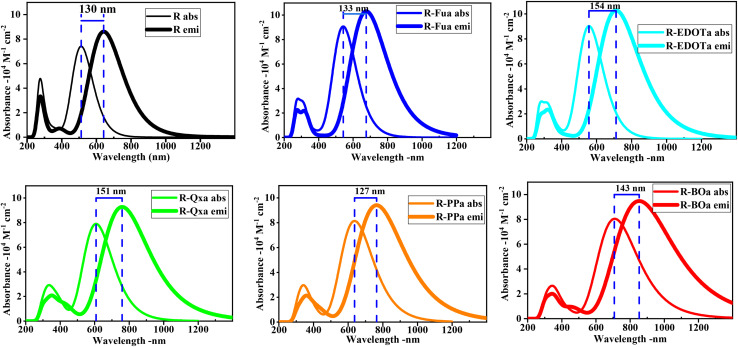
Emission and absorption spectra with the shift value between the maximum wavelength (*λ*_SS_) performed by employing TD-DFT/CAM-B3LYP/6-311G** under CPCM in DCM medium.

In order to calculate the emission spectra of the free dyes, we have optimized their first-lowest single excited state (S1) geometries using TD-DFT/CAM-B3LYP/6-311G** in DCM medium under CPCM. The emission spectra are illustrated in Fig. 5 and their corresponding parameters like the fluorescence energy (*E*_flu_), oscillator strength (*f*_e_), the maximum emission wavelength (*λ*_e_), the main transition contribution, and the Stokes Shifts (*λ*_ss_) between the maximum emission and the maximum absorption wavelengths are illustrated in Table 5. To compare the absorption and fluorescence characteristics of the free dyes, we have represented their absorption and emission spectra with their Stokes shift values in Fig. 6. The emission and absorption spectra have the same shape, with a narrow band in the ultraviolet domain and a large band in the vis and NIR domains, but the emission spectra are larger and significantly red-shifted than the absorption spectra. The oscillator strengths (*f*_em_) for the dyes R, R-Fua, R-EDOTa, R-Qx, R-PPa, and R-Boa, respectively, take 2.13, 2.55, 2.54, 2.29, 2.32, and 2.34 for the emission spectra than 1.83, 2.24, 2.23, 1.95, 2.01, and 1.99 for the absorption spectra. The maximum emission wavelengths (*λ*_em_) for the above dyes, respectively, take 642, 676, 711, 759, 764, and 853 nm, corresponding to Stokes shifts of approximately 130, 133, 154, 151, 127, and 143 nm, or 0.49, 0.45, 0.48, 0.41, 0.33, and 0.30 eV, respectively. Thess results show that R-Fua and R-EDOTa dyes with larger Stokes shifts (in eV) feature more flexible molecular architectures. In contrast, the small Stokes Shift values (in eV) of R-PPa and R-BOa dyes indicate a more rigid intramolecular and conjugated framework. On the other hand, the main transitions of the emission spectra are dominated by the LUMO → HOMO transition and their contributions range between 84 and 89%, which can be assigned to the ICT from A to D throughout Ap and π-spacer groups and/or to π* → π transitions.

**Table 5:** Emission parameters of free compounds were performed by employing TD-DFT/CAM-B3LYP/6-311G** in DCM medium under CPCM

Dyes	State	*λ*_e_ (nm)	*λ*_SS_ (nm)	Δ*E*_SS_ (eV)	*f*_e_	*E*_f_ (eV)	Main compositions (%)	*τ* (nm)
R	S_1_	642	130	0.49	2.13	1.93	L → H (89)	2.90
R-Fua	S_1_	676	133	0.45	2.55	1.83	L → H (85)	2.70
R-EDOTa	S_1_	711	154	0.48	2.54	1.74	L → H (88)	3.00
R-Qxa	S_1_	759	151	0.41	2.29	1.63	L → H (85)	3.79
R-PPa	S_1_	764	127	0.32	2.32	1.62	L → H (84); L → H−1 (10)	3.78
R-BOa	S_1_	853	143	0.29	2.34	1.45	L → H (85); L → H−1 (10)	4.68

It is known that the excited state lifetime (*τ*) represents the average time for the electron that can still be excited before emitting a photon. A high value of this parameter can ameliorate the electron transfer efficiency from the LUMO of the dye to the CB of the metallic semiconductor and consequently enhance the PCE. However, a short value of (*τ*) can increase the ability to emit light and decrease the PCE. The value of *τ* was calculated from the following expression:1
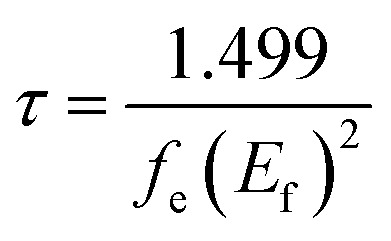
where *f*_e_ is the oscillator strength and *E*_f_ represents the fluorescence energy (in cm^−1^). The results are summarized in Table 5. The results show that the values of *τ* increase in the order: R-Fua (2.70 ns) < R (2.90 ns) < R-EDOTa (3 ns) < R-PPa (3.78 ns) < R-Qxa (3.79 ns) < R-BOa (4.68 ns), indicating that the designed dyes will be more efficient than the reference dye.

**Scheme 2:**
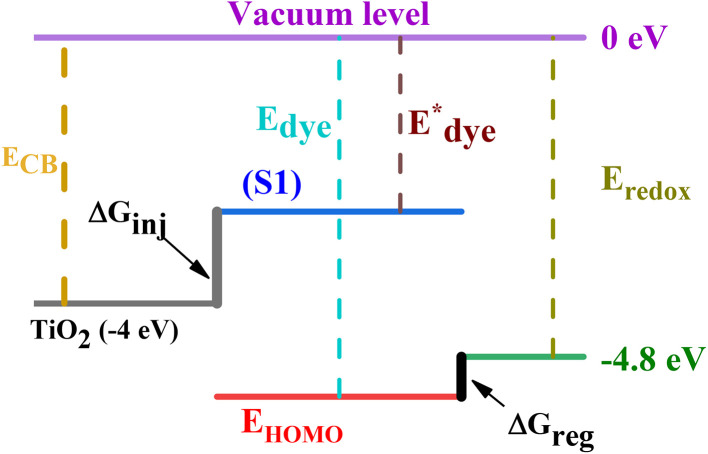
Representation of the driving force of electron injection and dye regeneration with energy arrangement of DSSC.

To confirm the spontaneity of the electron injection from the dyes to the CB of the semiconductor and the regeneration of the oxidized dyes by the electrolyte, the electronic injection driving force Δ*G*_inj_ and the driving force of regeneration Δ*G*_reg_ were calculated using the HOMO energy, excitation energy (*E*_ex_), conduction band energy (*E*_CB_ ∼ 4 eV), and potential redox of the electrolyte (*E*_redox_ ∼ 4.8 eV) by employing eqn (2) and (3) (see Scheme 2). The calculated values are illustrated in Table 4.2
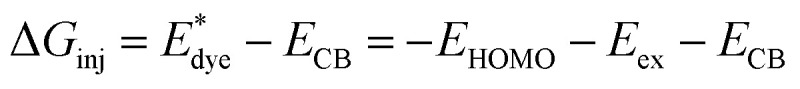

3 Δ*G*_reg_ = *E*_redox_ − *E*_HOMO_

The calculated values are all negative and range from −1.38 to −0.72 eV of Δ*G*_inj_, and from −0.24 to −0.08 eV of Δ*G*_reg_, indicating that the photoelectron can be injected into the conduction band (CB) of TiO_2_ and the regeneration of the oxidized dyes by the electrolyte can be realized spontaneously.

### 3.5. Molecular electrostatic potential of the free organic compound

**Fig. 7:**
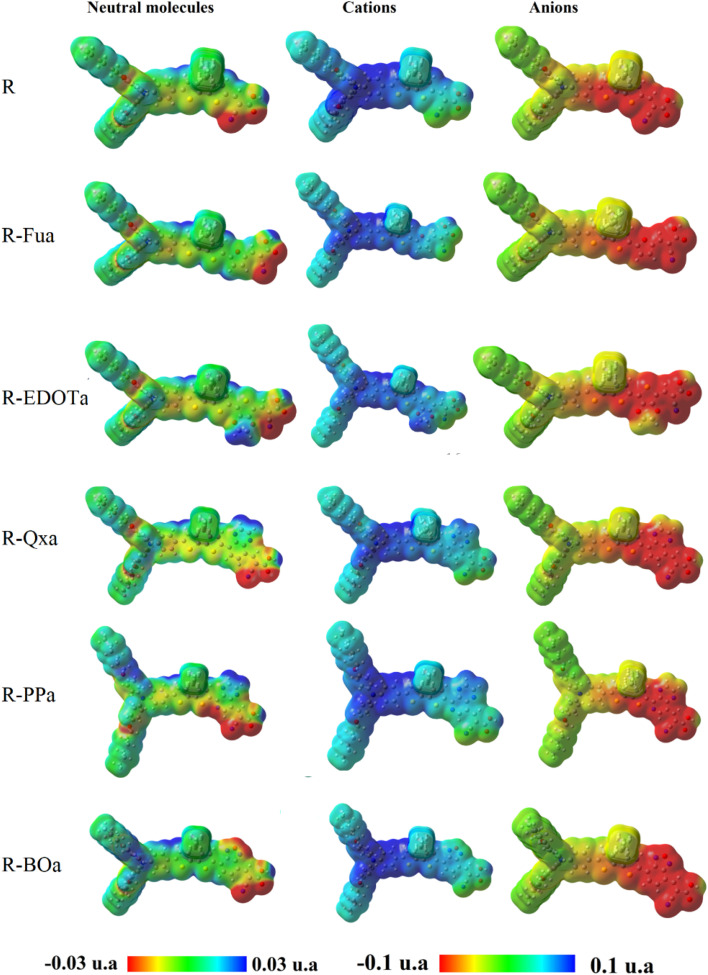
MEP maps of the neutral and charged dyes performed by DFT/B3LYP/6-311G** in DCM medium.

In general, the molecular electrostatic potential (MEP) of the free dye molecule in DSSC can be used to visualize the charge distribution, identify the primary site of electrophilic and nucleophilic reactivity, estimate the interaction mode with the metallic semiconductor, and estimate the electron transfer mechanism. For these purposes, we have calculated and illustrated in Fig. 7 the MEP maps of the neutral and charged dyes. In this figure, the MEPs are represented by colors as follows: red < orange < yellow < green < cyan < blue. For the cationic dyes, the MEPs show that the electropositive areas are concentrated primarily on the nitrogen (N) of the triphenylamine group. However, the electronegative regions for the anionic dyes are mainly distributed in the Ap and A groups. Consequently, the excited electron can come from the triphenylamine and move to the anchoring group throughout the π-bridge and auxiliary acceptor groups. For the neutral dyes, the principal electronegative areas are the nitrogen and the oxygen atoms of the –CN and C=O, respectively, in the cyanoacrylic acid group (CAA), while the electropositive areas are concentrated on the hydrogen atoms of the –COOH in the cyanoacrylic acid. These results show that the H and O atoms of the A group constitute a dipolar region that can be linked to the TiO_2_ semiconductor.

### 3.6. Electronic and absorption characteristics of adsorbed organic compounds

To study the electronic and optical characteristics of adsorbed organic compounds, we have chosen the basic cluster (TiO_2_)_9_ anatase (101)^72^ and optimized the dyes@(TiO_2_)_9_ clusters using DFT/B3LYP with two basis sets, LanL2DZ for the metallic atoms in semiconductor and 6-311G** for the non-metallic atoms in organic compound. Previous theoretical calculations confirmed that the adsorption of the organic dyes that contain the CAA as an anchor group is more stable with the bidentate chelating mode on the TiO_2_ surface.^71,73^ The energy of the free dye, (TiO_2_)_9_, and adsorbed dye@(TiO_2_)_9_ clusters are used to calculate the adsorption energy (*E*_ads_) of each dye using the following formula:

4 *E*_ads_ =  *E*_dye@(TiO_2_)_9__  −  *E*_dye_ − *E*_(TiO_2_)_9__

**Fig. 8:**
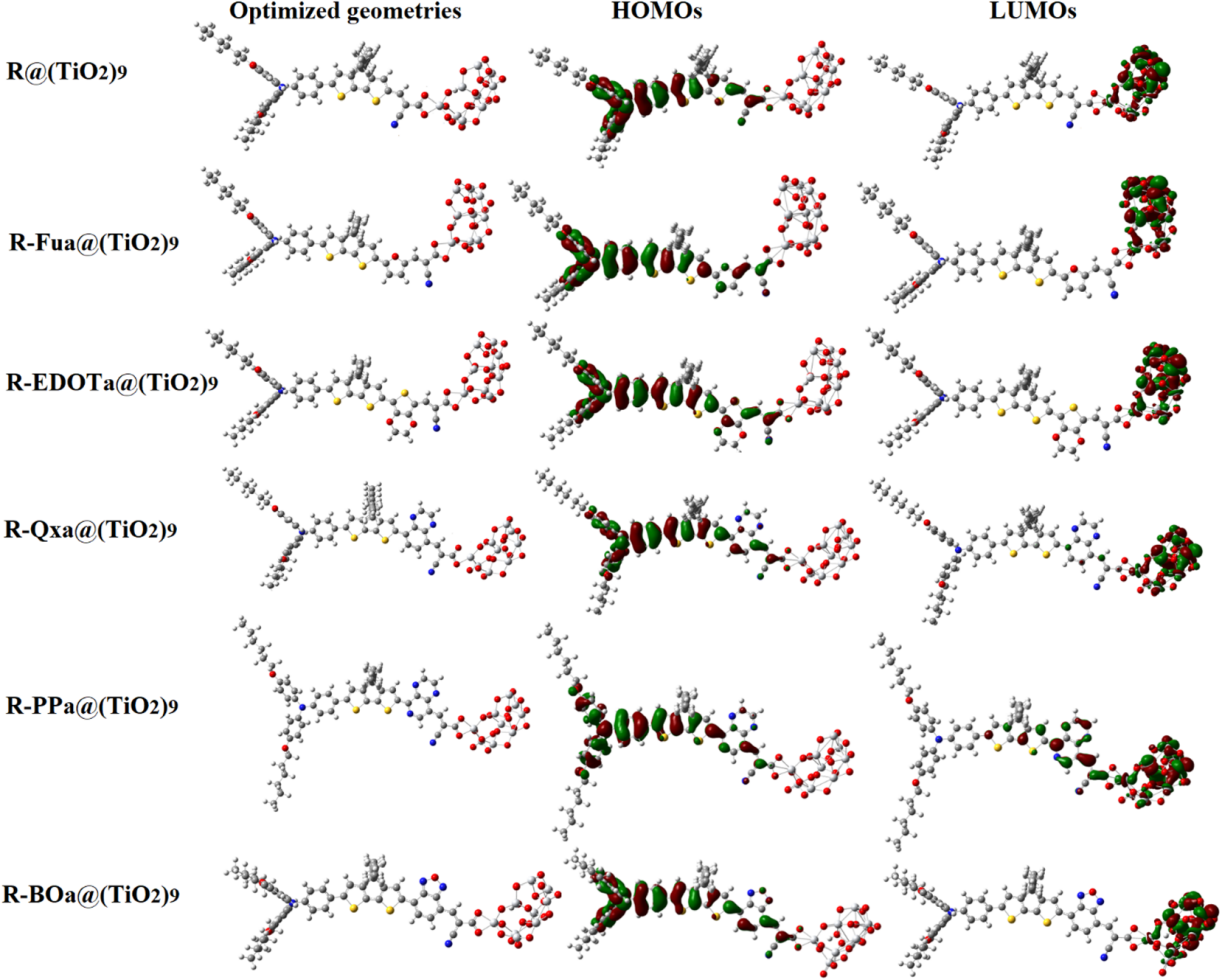
Optimized dye@(TiO_2_)_9_ clusters geometries with their HOMO, and LUMO distributions in DCM (isovalue of 0.02 au).

**Fig. 9:**
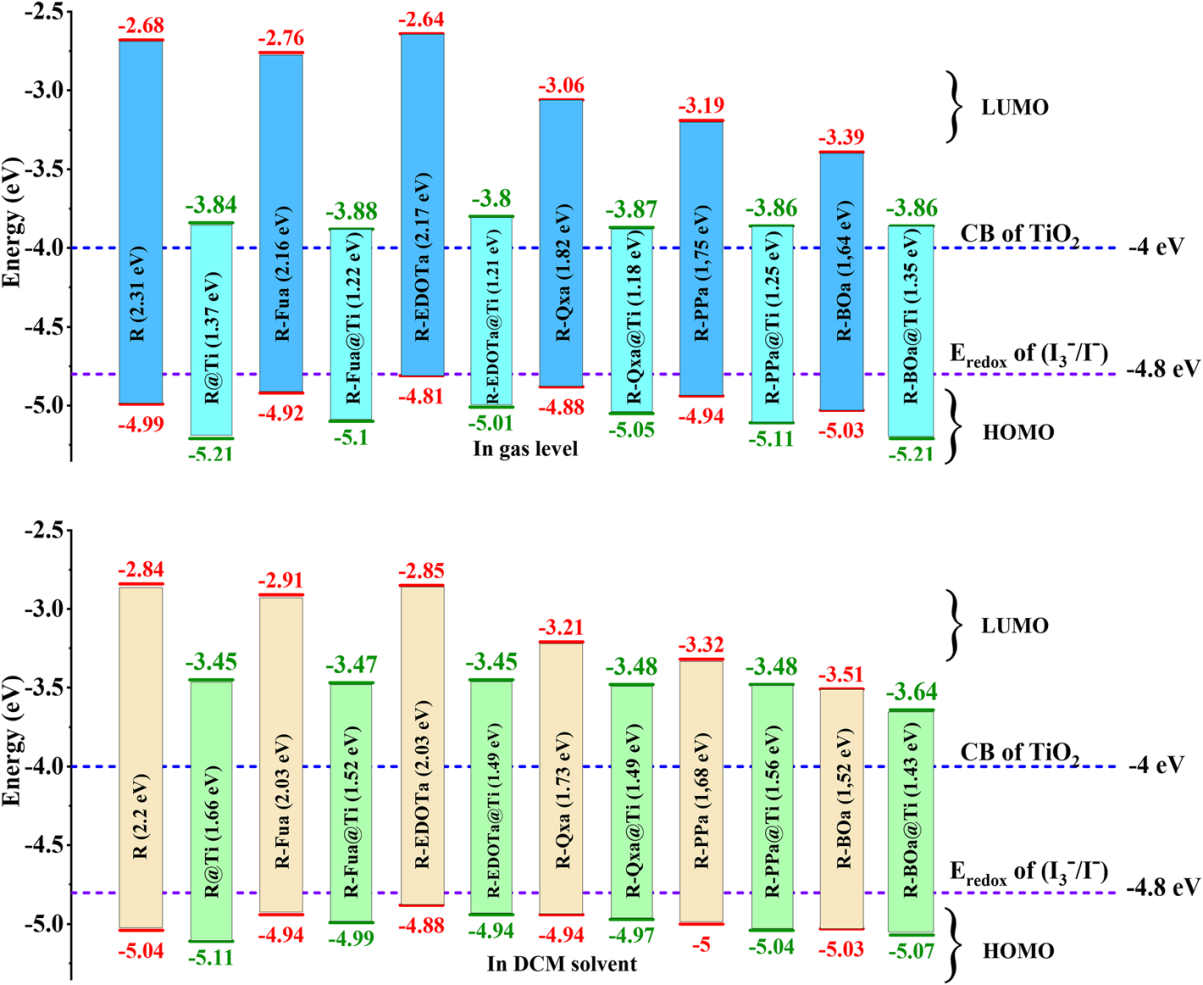
Energy diagram of the free and adsorbed dyes in the vacuum level and DCM medium.

The optimized dye@(TiO_2_)_9_ clusters along with the distribution and energy of their FMOs are illustrated in Fig. 8 and 9, the extracted parameters are illustrated in Table 6. The Ti–O bond lengths are between 2.030 and 2.075 Å. These values show a good agreement with the Ti–O bond lengths (ranging from 1.934 and 1.98 Å) of the bulk TiO_2_. On the other hand, the adsorption energies of the studied dyes are all negative and take the values between −1.08 and −0.85 eV in DCM and between −1.60 and −1.52 eV in vacuum level, indicating that the adsorption of dye molecules onto the TiO_2_ surface is exothermic.^74,75^ These large negative values with short Ti–O bond lengths indicate that the adsorption of our investigated dyes occurs spontaneously with a chemical process, confirming their strong adsorption on the metallic semiconductor surface.

**Table 6:** Ti–O bond lengths (Å). Total ground state energy of free dyes, (TiO_2_)_9_ and dyes@(TiO_2_)_9_ clusters. LUMO, HOMO, Gap, and adsorption energies (all energies in eV)

Dyes@(TiO_2_)_9_	Media	Ti–O	Ti–O	*E*_dyes_	*E*_(TiO_2_)_9__	*E*_dye/s@TiO2)9_	*E*_HOMO_	*E*_LUMO_	*E*_Gap_	*E*_ads_
R@(TiO_2_)_9_	Vacuum	2.031	2.070	−91002.950	−51129.921	−142134.439	−5.21	−3.84	1.37	−1.57
R-Fua@(TiO_2_)_9_		2.036	2.074	−97231.53	−51129.921	−148363.01	−5.10	−3.88	1.22	−1.56
R-EDOTa@(TiO_2_)_9_		2.030	2.065	−112221.50	−51129.921	−163353.02	−5.01	−3.80	1.21	−1.60
R-Qxa@(TiO_2_)_9_		2.040	2.074	−103416.30	−51129.921	−154547.76	−5.05	−3.87	1.18	−1.54
R-PPa@(TiO_2_)_9_		2.072	2.041	−102782.82	−51129.921	−153914.28	−5.11	−3.86	1.25	−1.54
R-BOa@(TiO_2_)_9_		2.047	2.075	−102284.20	−51129.921	−153415.65	−5.21	−3.86	1.35	−1.52
R@(TiO_2_)_9_	DCM	2.031	2.070	−91003.548	−51133.617	−142138.015	−5.11	−3.45	1.66	−0.85
R-Fua@(TiO_2_)_9_		2.036	2.074	−97232.14	−51133.617	−148366.81	−4.99	−3.47	1.52	−1.06
R-EDOTa@(TiO_2_)_9_		2.030	2.065	−112222.24	−51133.617	−163356.94	−4.94	−3.45	1.49	−1.08
R-Qxa@(TiO_2_)_9_		2.040	2.074	−103416.94	−51133.617	−154551.58	−4.97	−3.48	1.49	−1.03
R-PPa@(TiO_2_)_9_		2.041	2.072	−102783.50	−51133.617	−153918.14	−5.04	−3.48	1.56	−1.03
R-BOa@(TiO_2_)_9_		2.047	2.075	−102284.83	−51133.617	−153419.48	−5.07	−3.64	1.43	−1.03

The HOMO orbitals of the adsorbed dyes are strongly localized on the D group and slightly distributed over the π-spacer group, the LUMOs are located entirely on the semiconductor cluster, however. Consequently, the photoelectrons created in the D group can be spontaneously transported throughout the π and Ap groups and injected *via* the A group into the metallic semiconductor. All HOMO energies of the adsorbed organic compounds are positioned under the redox potential of the (I^−^_3_/I^−^) couple and their values of the free organic compounds, indicating the spontaneity of the regeneration process by the iodide ions (I^−^) in the electrolyte. However, their LUMO energies are positioned above the CB of the metallic semiconductor and take the values between −3.8 and −3.87 eV in vacuum and between −3.45 and −3.64 eV in DCM. In addition, the gap energy of the adsorbed dyes becomes narrower than that of the free dyes. Consequently, exciting electrons from the HOMO to the LUMO by absorbing photons will be easier, and their UV-vis absorption spectra will become broader and shift to the red and NIR domains compared to the free dyes.

**Fig. 10:**
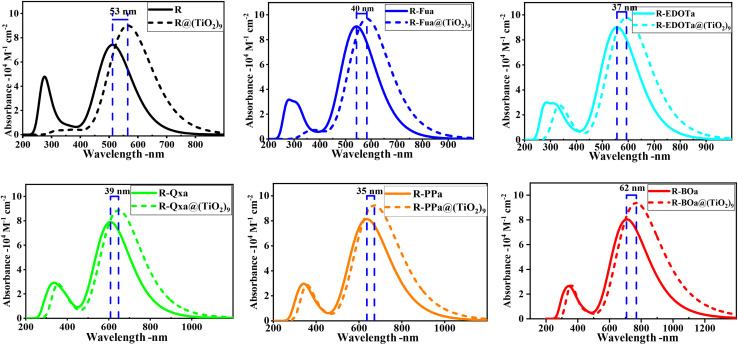
Comparison between the UV-vis absorption spectra of the dyes@(TiO_2_)_9_ systems and free organic compounds in DCM medium.

To reduce the computational cost for calculating the UV-vis absorption spectra of dyes@(TiO_2_)_9_ clusters, we have replaced –C_6_H_13_ by –CH_3_. These new clusters were reoptimized and considered to extract the absorption spectra by employing TD-DFT/CAM-B3LYP in DCM medium using two basis sets, LanL2DZ for the metallic atoms in semiconductor and 6-311G** for the non-metallic atoms in organic compound. The UV-vis absorption spectra of the dyes@(TiO_2_)_9_ clusters with their Stokes shift compared to the free dyes are illustrated in Fig. 10, and their optical parameters are calculated and illustrated in Tables 7 and SD4.†

**Table 7:** Optical factors of the adsorbed dyes@(TiO_2_)_9_ systems in DCM medium

Dyes@(TiO_2_)_9_	State	*λ*_max_ (nm)	*E*_ex_	*f*	Main compositions (%)
R@(TiO_2_)_9_	S_1_	556	2.19	2.22	H → L+1 (58); H−1 → L+1 (21)
R-Fua@(TiO_2_)_9_	S_1_	583	2.13	2.39	H → L+1 (46); H−1 → L+1 (25); H → L (11)
R-EDOTa@(TiO_2_)_9_	S_1_	593	2.09	2.42	H → L (32); H → L+1 (27); H−1 → L (15); H−1 → L+1 (13)
R-Qxa@(TiO_2_)_9_	S_1_	647	1.92	2.19	H → L (67); H−1 → L (23)
R-PPa@(TiO_2_)_9_	S_1_	672	1.84	2.28	H → L (68); H−1 → L (25)
R-BOa@(TiO_2_)_9_	S_1_	769	1.61	2.31	H → L (69); H−1 → L (25)

As illustrated in Fig. 10, the absorption spectra of the adsorbed organic compound are shifted to the red region, broadened, and exhibit higher oscillator strengths than the free organic compounds. The shift of the *λ*_max_ enhances in the order R@(TiO_2_)_9_ (556 nm) < R-Fua@(TiO_2_)_9_ (583 nm) < R-EDOTa@(TiO_2_)_9_ (593 nm) < R-Qxa@(TiO_2_)_9_ (647 nm) < R-PPa@(TiO_2_)_9_ (672 nm) < R-BOa@(TiO_2_)_9_ (769 nm), indicating that the introduction of Fu, EDOT, Qx, PP, and BO as auxiliary electron-withdrawing groups leads to broaden the UV-vis absorption spectra and shift them to the red domain than the R@(TiO_2_)_9_ cluster. These findings suggest that the dyes interact strongly with the semiconductor, making them suitable as sensitizing dyes in DSSCs.

### 3.7. Reorganization energies (REs) and chemical reactive parameters (CRPs)

Based on the electron transfer theory, the charge transfer rate k_CT_ is related to the reorganization energy *λ* (RE) by expression (5).^76^ In this expression, *A*, *k*_B_, and *T* represent a pre-exponential factor, the Boltzmann constant (8.6173 × 10^−5^ eV K^−1^), and absolute temperature (300 K), respectively, which are considered as constants. Therefore, a small value of RE is beneficial to accelerate the charge transfer rate and consequently improve *J*_SC_.5
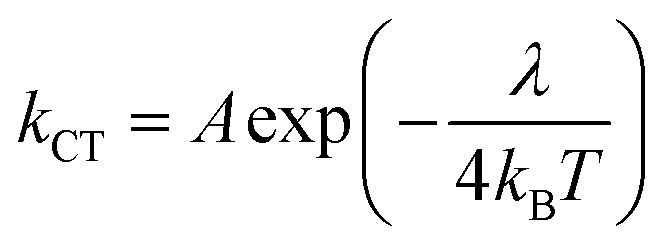


The RE of the studied dyes is taken as the sum of the hole reorganization energy *λ*_h_ (HRE) and the electron reorganization energy *λ*_e_ (ERE), which are calculated using eqn (6) and (7).^58,77^

6 *λ*_h_ = (*E*_n_^−^ − *E*_a_) + (*E*^0^_a_ − *E*_a_)

7 *λ*_h_ = (*E*_n_^+^ − *E*_c_) + (*E*^0^_c_ − *E*_n_)

where *E*_n_, *E*_c_, and *E*_a_ are the total energies of the optimized neutral, cationic, and anionic dyes, respectively. *E*_n_^+^/ *E*_n_^−^ represent the cationic/anionic energies calculated without optimization by removing/adding one electron from/to the neutral dyes, respectively. 
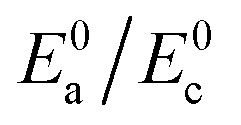
 represent the total energies of the neutral dyes calculated without optimization by removing/adding one electron from/to the anionic/cationic dyes, respectively. The calculated results are tabulated in Table 8.

**Table 8:** Vertical (v) and adiabatic (a) CRPs (*I*, *A*, *h*, *ω*, and *ω*^+^) and REs (*λ*_h_, *λ*_h_, and *λ*_tot_) in vacuum level and DCM medium (all values are in eV)

Molecules	Levels	*I*_v_	*I*_a_	*E*_v_	*E*_a_	*h*_v_	*h*_a_	*ω*_v_	*ω*_a_	*ω*_v_^+^	*ω*_a_^+^	*λ*_h_	*λ*_e_	*λ*_tot_
R	DCM	5.011	4.900	2.889	3.021	1.061	0.939	7.353	8.350	5.511	6.487	0.207	0.263	0.471
R-Fua		4.907	4.784	2.927	3.050	0.990	0.867	7.749	8.848	5.914	6.998	0.212	0.239	0.451
R-EDOTa		4.851	4.718	2.880	3.023	0.986	0.848	7.581	8.838	5.771	7.009	0.233	0.280	0.512
R-Qxa		4.896	4.790	3.244	3.351	0.826	0.720	10.027	11.514	8.095	9.569	0.189	0.211	0.400
R-PPa		4.955	4.850	3.365	3.466	0.795	0.692	10.884	12.492	8.903	10.500	0.191	0.201	0.392
R-BOa		4.983	4.870	3.543	3.645	0.720	0.613	12.620	14.797	10.579	12.745	0.191	0.202	0.393
R	Vacuum	5.957	5.825	1.536	1.755	2.211	2.035	3.176	3.529	1.578	1.888	0.236	0.434	0.670
R-Fua		5.818	5.684	1.652	1.837	2.083	1.924	3.349	3.676	1.741	2.036	0.320	0.372	0.692
R-EDOTa		5.685	5.537	1.583	1.792	2.051	1.873	3.219	3.586	1.659	1.988	0.275	0.414	0.689
R-Qxa		5.760	5.648	2.000	2.142	1.880	1.753	4.004	4.327	2.299	2.599	0.209	0.284	0.493
R-PPa		5.819	5.708	2.138	2.275	1.841	1.717	4.300	4.641	2.541	2.860	0.216	0.276	0.492
R-BOa		5.913	5.800	2.315	2.450	1.799	1.675	4.704	5.079	2.872	3.226	0.207	0.281	0.487

We can see from this Table that the total RE decreases in the order R-EDOTa (0.512 eV) > R (0.471 eV) > R-Fua (0.451 eV) > R-Qxa (0.400 eV) > R-BOa (0.393 eV) > R-PPa (0.392 eV) in DCM medium, and follows the order R-Fua (0.692 eV) > R-EDOTa (0.689 eV) > R (0.670 eV) > R-Qxa (0.493 eV) > R-PPa (0.492 eV) > R-BOa (0.487 eV) in vacuum, indicating that R-Qxa, R-PPa, and R-BOa have approximately the same minimum values of the RE. Consequently, these three dyes will generate high values of *J*_SC_. The ERE decreases in the order R-EDOTa (0.280 eV) > R (0.263 eV) > R-Fua (0.239 eV) > R-Qxa (0.211 eV) > R-BOa (0.202 eV) > R-PPa (0.201 eV) in DCM medium, and follows the order R (0.434 eV) > R-EDOTa (0.414 eV) > R-Fua (0.372 eV) > R-Qxa (0.284 eV) > R-BOa (0.281 eV) > R-PPa (0.276 eV) in vacuum, indicating that R-Qxa, R-PPa, and R-BOa represent the best organic compounds which can function as electron transporting compounds. The HRE decreases as follows: R-EDOTa (0.233 eV) > R-Fua (0.212 eV) > R (0.207 eV) > R-PPa = R-BOa (0.191 eV) > R-Qxa (0.189 eV) in DCM medium, and follows the order R-Fua (0.320 eV) > R-EDOTa (0.275 eV) > R (0.236 eV) > R-PPa (0.216 eV) > R-Qxa (0.209 eV) > R-BOa (0.207 eV) in vacuum, indicating that R-Qxa, R-PPa, and R-BOa represent the best organic compounds which can act as hole transporting compounds.

**Fig. 11:**
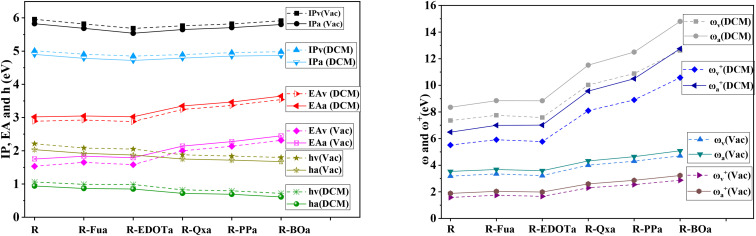
Plot of adiabatic (a) and vertical (v) chemical reactive parameters of the investigated dyes in vacuum level (*V*ac) and DCM medium calculated by employing DFT/B3LYP/6-311G**.

The *E*_n_, *E*_c_, and *E*_a_ energies are also used to calculate IP and EA using eqn (8) and (9). After that, IP and EA were used to calculate *h*, *ω*, and *ω*^+^ using eqn (10)–(12). The CRPs were calculated in both vertical and adiabatic ways (show figure SD1†), the calculated results are summarized in Table 8 and illustrated in Fig. 11.

8 *I* = *E*_c_ − *E*_n_

9 *A* = *E*_n_ − *E*_a_

10 *h* = (IP − EA)/2

**Fig. 12:**
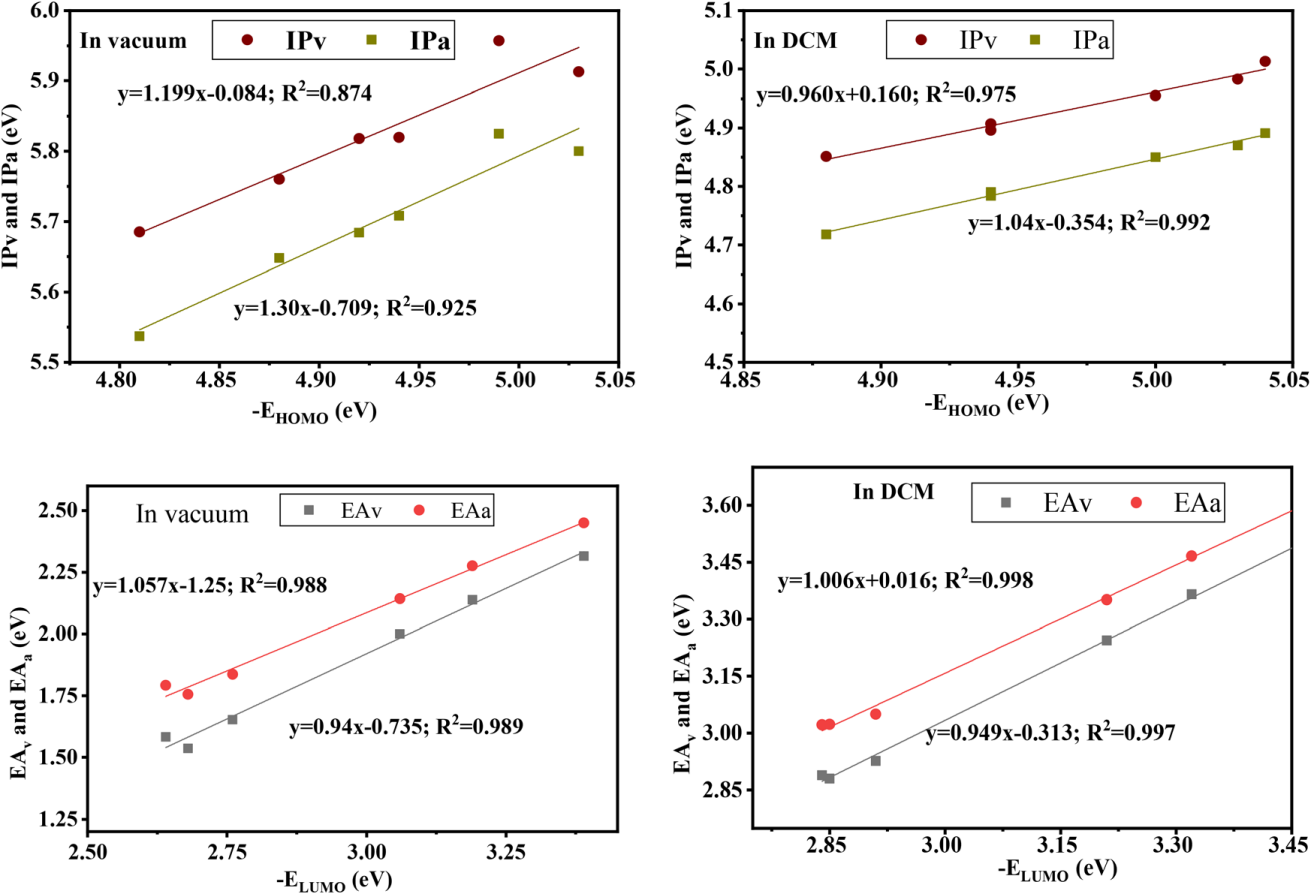
Correlation between IP and −*E*_HOMO_ and between EA and −*E*_LUMO_ in the vacuum level and DCM medium calculated by employing DFT/B3LYP/6-311G**.

11
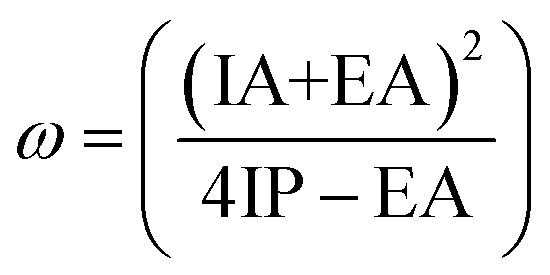
12
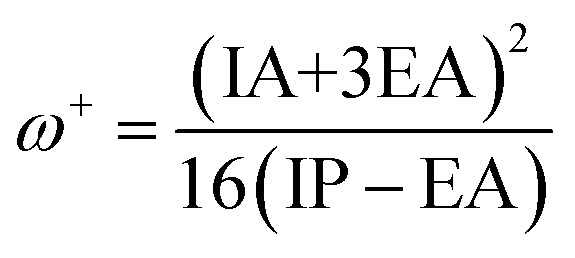
IP and EA of dye molecules represent the crucial parameters that can give more information about charge injection. A large value of EA and a small value of IA are desired to ameliorate the charge injection of dye molecules. As illustrated in Table 8 and Fig. 11, the smallest IP value is taken from R-EDOTa followed by R-Qxa < R-Fua < R-PPa < R-BOa < R, and the higher EA value is obtained for R-BOa followed by R-PPa > R-Qxa > R-Fua > R-EDOTa > R. Indeed, the smallest IP values are obtained in adiabatic way and DCM medium, and the larger EA values are obtained in vertical way and DCM medium. These results indicate that incorporating an Ap group in the reference dye reduces IP and enhances EA values. Consequently, the designed dyes will be more efficient than the reference dye. Furthermore, the correlation between IP and −*E*_HOMO_ and between EA and −*E*_LUMO_ can be realized by fitting plots, as illustrated in Fig. 12.^78,79^ We can see from this figure the good correlations between the mentioned parameters, and this correlation is better in DCM than in the vacuum level. Chemical hardness (*h*) is another crucial chemical parameter that can provide more information about dyes' ICT resistance. A small chemical hardness value is preferred to decrease the ICT resistance of the dye and consequently enhance its efficiency. The calculated results show that this parameter takes a smaller value in DCM medium and for the adiabatic way than in the vacuum level and vertical way. The smallest value of *h* is taken from the R-BOa dye, followed by R-PPa < R-Qxa < R-EDOTa < R-Fua < R, suggesting that the R-BOa is the best compound in the series, which can generate high *J*_SC_ and improve the PCE. The *ω* and *ω*^+^ parameters can also provide more information about the performance of the dye; a large value of these parameters is desired to enhance the PCE. The *ω* and *ω*^+^ values are smaller in the DCM medium for the adiabatic than in the vacuum level and vertical way. The smallest values are obtained for R-BOa dye, followed by R-PPa > R-Qxa > R-Fua > R-EDOTa > R, indicating that R-BOa is the best dye in the series. Indeed, the reactive chemical parameters are all better for the designed dyes than the reference dye, confirming that inserting an Ap can enhance the PCE.

### 3.8. Hole electron investigation

To investigate the ICT properties of the studied organic compounds, the charge density difference CDD (Δ*ρ*(*r*)), the electron and hole centroid with the distance between their centers (*D* index), *H*_CT_ index, *t* index, electron–hole overlap function *S*_r_, and the hole–electron attractive Coulomb energy (*E*_C_) were calculated as referred in the literature (as shown in eqn SD1–SD6†).^59,60,80–82^ Indeed, we calculated the number of electrons (NET) that can be transferred between different groups of dyes, interfragmentary electron redistribution (IFER), The contribution of HOMO and LUMO in hole and electron, local excitation proportion LE (%), and intrinsic CT proportion CT (%) for the studied organic compounds upon S0 → S1 excitation.

**Fig. 13:**
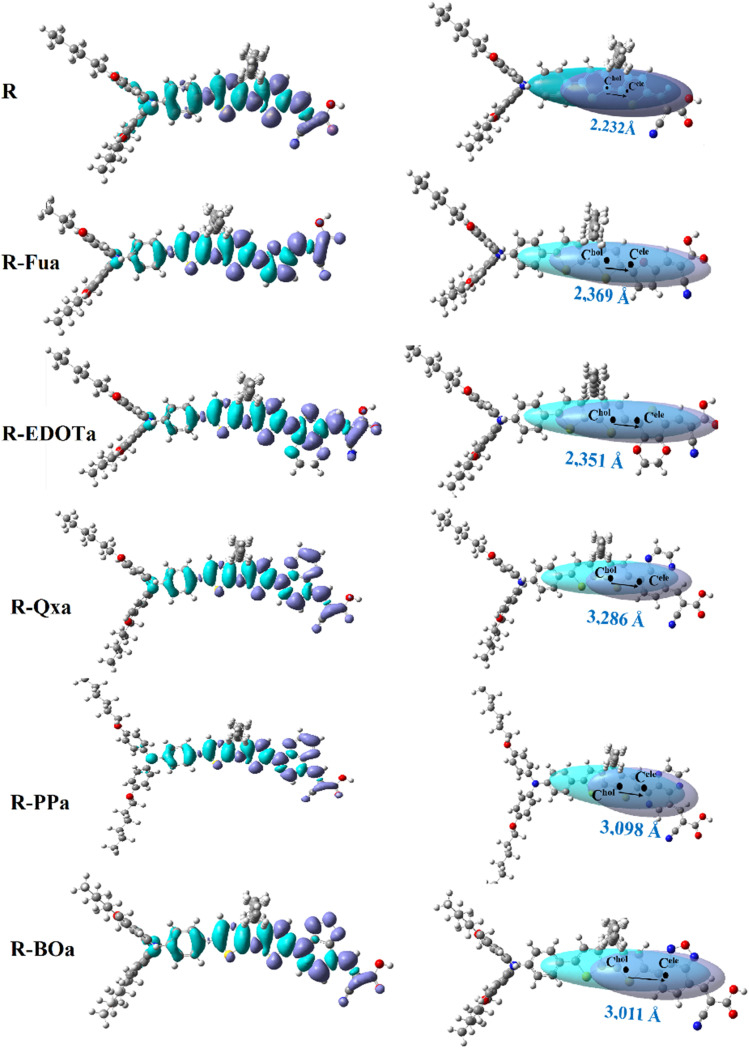
CDD (left) and hole electron centroid with their centers and D index (right) of the free dyes upon S_0_ → S_1_ excitation in DCM medium (iso density 0.0004 au).

**Fig. 14:**
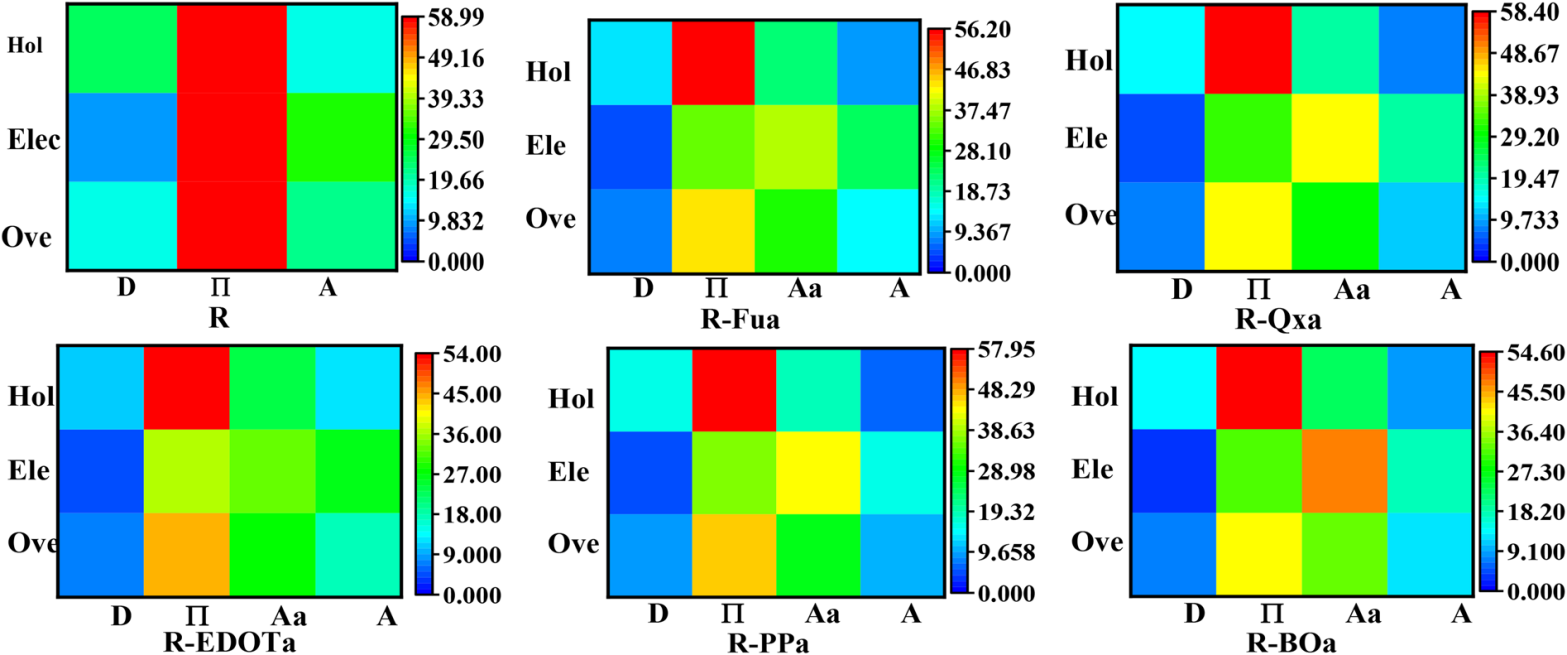
Heat maps that represent the percentage contribution by different groups to holes (Hol), electrons (Ele), and the hole–electron overlap (Ove).

The CDD and hole electron centroids upon S0 → S1 excitation are illustrated in Fig. 13, and the percentage contribution by each group to electrons and holes is summarized in Table 9 and illustrated as heat maps in Fig. 14.

**Table 9:** Contribution (in %) of the different groups to holes and electrons with their difference and the electron–hole overlaps for each compound in DCM medium

Dyes	Groups	Contribution to hole	Contribution to electron	Hole–electron overlapping	Difference
R	D	25.16	9.71	15.63	−15.45
	Π	58.74	58.99	58.86	0.25
	A	16.10	31.30	22.45	15.20
R-Fua	D	12.02	4.90	7.67	−7.12
	Π	53.83	38.71	45.65	−15.12
	Aa	21.34	26.00	23.55	4.36
	A	12.82	30.39	19.74	17.58
R-EDOTa	D	10.75	4.83	7.20	−5.93
	Π	53.88	36.61	44.41	−17.27
	Aa	23.59	32.66	27.76	9.07
	A	11.78	25.91	17.47	14.12
R-Qxa	D	14.20	4.29	7.81	−9.91
	Π	58.38	32.97	43.87	−25.41
	Aa	19.91	43.16	29.32	23.24
	A	7.50	19.58	12.12	12.08
R-PPa	D	16.20	4.78	8.80	−11.43
	Π	57.95	36.28	45.85	−21.67
	Aa	18.98	43.43	28.71	24.45
	A	6.87	15.52	10.33	8.64
R-BOa	D	13.57	3.76	7.14	−9.81
	Π	54.55	31.21	41.26	−23.34
	Aa	22.86	46.94	32.76	24.09
	A	9.02	18.09	12.77	9.07

For R dye, the hole is primarily distributed on the π-D groups (cyan lobes), and their contributions take 58.74% and 25.16% with red and green colors in the heat map, respectively. The electron is primarily distributed on the π-Ap groups (purple lobes); their contributions take 58.99% and 31.30% and appear in the heat map with red and green colors, respectively. The hole–electron overlap is primarily distributed over the π-Ap groups; their contributions take 58.86% and 22.45% and appear in red and green colors in the heat map, respectively. These results show that the D group contributes more to the hole than the electron, with a negative difference (−15.45%), confirming its donating ability. In contrast, the A group contributes more to the electron than the hole with a positive difference (15.20%), confirming its electron-accepting ability. On the other hand, the contribution of the π group is the same, with a small positive difference (0.25%), indicating that this group acts as a bridging group.

For the designed D-π-Ap-A organic compounds, the holes are primarily distributed on the π group (cyan lobes), and their contributions are in the range of 53.83 to 58.38%, which appear in the heat maps in red. The electrons are mainly distributed on the π, Ap, and A groups, and their contributions range from 31.21 to 38.71% for the π group which appears in the heat map in green, range from 26 to 46.94% for the Ap group and appears in the heat map in green, yellow or orange, and range from 15.52 to 30.39% for the A group which appear in the heat map in cyan or green colors. The hole–electron overlap occurs mainly in the π and Ap groups, their contributions range from 41.26 to 45.85% for the π group, which appears in the heat map in yellow or orange, and from 23.55 to 32.76% for the Ap group, which appears in the heat map in green. Indeed, the D group contributes more to the holes than electrons; its difference ranges between −7.12 and −11.43%, confirming its electron donor character. The π group contributes more to the holes than electrons; its difference ranges between −15.12 and −25.41%, indicating that this group acts as an electron donor group. For the Ap group, the difference ranges from 4.36 to 24.45%, indicating that this group contributes more to the electrons than holes and consequently acts as an electron-withdrawing group. The A group contributes more to the electrons than holes; its difference ranges from 8.64 to 17.58%, confirming its electron-withdrawing character. From the above discussions, we can see that the insertion of the Ap group ameliorates the separation charge between different groups of the designed dyes and enhances their performances.

The charge transfer parameters, including *D* index, *H*_CT_, *t*, *S*_r_, and *E*_C,_ are calculated and illustrated in Table 10. The *D* index for the designed D-π-Ap-A organic compounds becomes more important than the reference dye and its values range from 2.232 to 3.286 Å, which are larger than the C–C (1.54 Å) and C=C bonds (1.43 Å), indicating that the electronic transition occurs by ICT. The values of *H*_CT_ are larger than the *D* index values, indicating the broad distribution of holes and electrons. The negative values of the *t* index and the large values of the *S*_r_ and *E*_CT_ show that holes and electrons are not separated, and the overlap between them is significant.

**Table 10:** ICT's parameters of the studied organic compounds were determined by employing hole–electron investigation

Dyes	Transition	*D* (Å)	*H*_CT_ (Å)	*t* (Å)	*S*_r_	*E*_C_ (eV)
R	S0 → S1	2.232	4.141	−1.909	0.740	3.752
R-Fua	S0 → S1	2.369	4.746	−1.488	0.750	3.776
R-EDOTa	S0 → S1	2.351	4.738	−2.387	0.753	3.712
R-Qxa	S0 → S1	3.286	4.528	−1.242	0.713	3.307
R-PPa	S0 → S1	3.098	4.924	−1.439	0.700	3.393
R-BOa	S0 → S1	3.011	3.543	−1.532	0.716	3.396

**Fig. 15:**
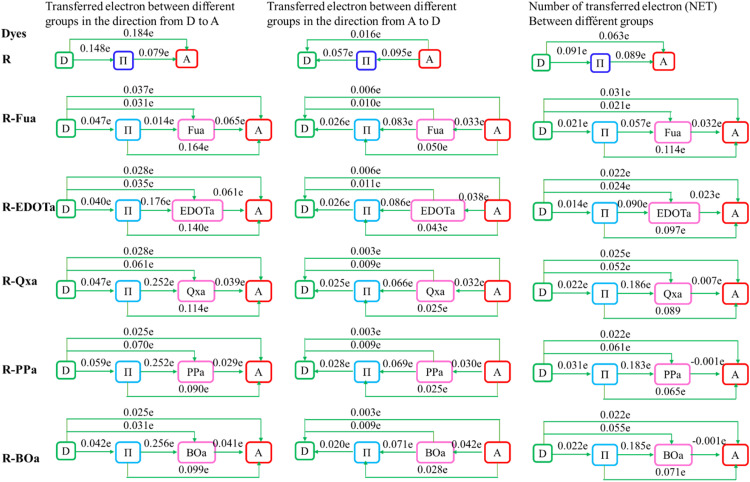
The transferred electron number (NET) between D, π, Ap, and A groups for the S_0_ → S_1_ excitation.

Calculating the NET and IFER can help us to identify where the electrons are produced and where they will move upon S0 → S1 excitation. These two parameters are determined and illustrated in Fig. 15 and Table 11.

**Table 11:** IFER (in e) for different groups. Participation of holes and electrons by different FMOs. Intrinsic CT (%) and LE (%) for the studied organic compounds during the excitation S_0_ → S_1_

Compounds	Groups				Participation of electrons				Participation of hole (%)				LE (%)	CT (%)
	D	π	Ap	A	H−1	H	L	L+1	H−1	H	L	L+1		
R	0.0244	0.3465	—	0.0504	0	0	95	4	30	67	0	0	42	58
R-Fua	0.0059	0.2084	0.0555	0.0390	0	0	91	7	28	66	0	0	31	69
R-EDOTa	0.0052	0.1972	0.0770	0.0305	0	0	93	6	25	70	0	0	31	69
R-Qxa	0.0061	0.1925	0.0860	0.0147	0	0	93	4	24	71	0	0	30	70
R-PPa	0.0077	0.2102	0.0824	0.0107	0	0	94	4	25	71	0	0	31	69
R-BOa	0.0051	0.1703	0.1073	0.0163	0	0	95	3	26	69	0	0	30	70

For the R dye, the NET shows that the D group can push 0.184 and 0.079 to the π and A groups and pull from them 0.057 and 0.016*e*, respectively. The π group can pull 0.148 and 0.095*e* from the D and A groups and give them 0.057 and 0.184*e*, respectively. Indeed, the A group can pull 0.079 and 0.184*e* from the D and π groups and give them 0.016 and 0.095, respectively. Therefore, D pushes 0.0154*e*, π pushes 0.002*e*, and A pulls 0.152.

For the R-Fua, R-EDOTa, R-Qxa, R-PPa, and R-BOa designed dyes, the D group can push 0.021, 0.014, 0.022, 0.031, and 0.022*e* to the π group, push 0.021, 0.024, 0.052, 0.061, and 0.055*e* to the Ap group, and push 0.031, 0.022, 0.025, 0.022, and 0.022*e* to the A group, respectively. Therefore, it pushes in total 0.073*e*, 0.060, 0.099, 0.114, and 0.099*e*, respectively. The π group can pull 0.021, 0.014, 0.022, 0.031, and 0.022*e* from the D group, push 0.057, 0.090, 0.186, 0.183, and 0.185*e* to the Ap group, and push 0.114, 0.097, 0.089, 0.065, and 0.071*e* to the A group, therefore, it pushes in total 0.150, 0.173, 0.252, 0.217, and 0.234*e*, for the above dyes respectively. The Ap group pulls 0.021, 0.024, 0.052, 0.061, and 0.055*e* from the D group, 0.057, 0.090, 0.186, 0.183, and 0.185*e* from the π group, and pushes 0.032, 0.023, 0.007, −0.001, and −0.001*e* to the A group, therefore, it pulls 0.046, 0.091, 0.231, 0.245, and 0.241, for the above dyes respectively. The A group pulls 0.031, 0.022, 0.025, 0.022, and 0.022*e* from the D group, 0.114, 0.097, 0.089, 0.065, and 0.071 *e* from the π group, and 0.032, 0.023, 0.007, −0.001, and −0.001*e* from the Ap group, therefore it pulls 0.177, 0.142, 0.121, 0.086, and 0.092, for the above dyes respectively. These results show that the insertion of the Ap group ameliorates the ICT between different groups and enhances their performances.

The results in Table 11 show that the IFER of the different groups ranges from 0.0051 to 0.0244*e* for D, from 0.0305 to 0.0504*e* for A, from 0.0555 to 0.1073*e* for Ap, and from 0.1703 to 0.3465*e* for π. These results show that the π group acts as a redistributor group that can push and pull electrons, confirming its bridging character. The intrinsic CT (%) ranges from 58 to 70%, these values are more important than the LE (%) values, which are between 30 and 42%. The larger CT (%) values than LE (%) confirm that the electronic transition during S_0_ → S_1_ excitation occurs primarily through the ICT. On the other hand, the holes are primarily focused by the contribution of HOMO (between 66 and 71%) followed by the contribution of HOMO-1 (ranging from 24 to 30%). In comparison, the electrons are dominated by the contribution of the LUMO (ranging from 91 to 95%). So, the electronic transition during S_0_ → S_1_ excitation occurs primarily by H → L transition followed by the H−1 → L transition, confirming the ICT excitation for the designed dyes.

### 3.9. Predicted photovoltaic parameters

#### 3.9.1. Predicted *J*_SC_

To estimate the *J*_SC_ theoretically, the LHE should be first predicted, this parameter can be predicted using eqn (13).^27,83^ In this equation, *ε*(*λ*) represents the molar absorption value extracted by employing TD-DFT/CAM-B3LYP/6-311G**, *d* is the experimental film thickness of TiO_2_ (5 μm), and *ρ* is the experimental dye loading amount on the TiO_2_ surface (1.8 × 10^−8^ mol^−1^ cm^−2^ μm^−1^).^34^ The theoretical LHE(*λ*) curves were performed and illustrated in Fig. 16.13
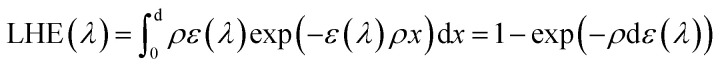


**Fig. 16:**
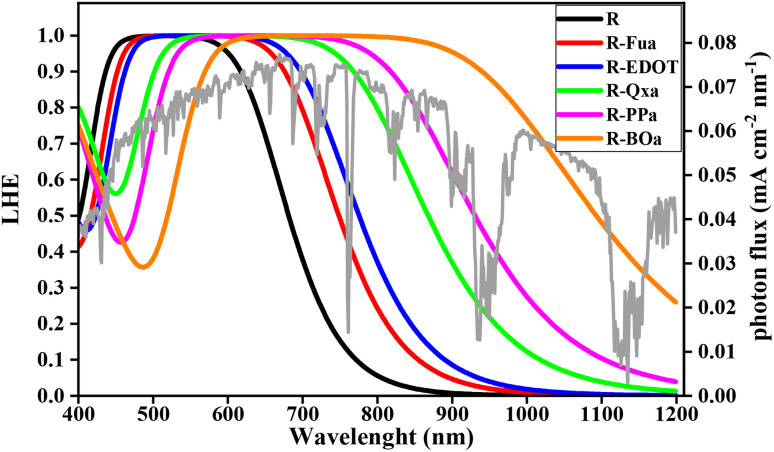
LHE curves of the studied dyes, solar spectrum (grey line).

The predicted LHE(*λ*) curves for the studied organic compounds appear substantial and encompass the entire Vis and NIR domains. Indeed, incorporating Fu, EDOT, Qx, PP, and BO groups as Ap groups enhances the light-harvesting capability. Consequently, the designed D-π-Ap-A dyes are expected to produce high *J*_SC_ and improve the PCE in DSSC.

After simulating the LHE(*λ*), we can estimate the *J*_SC_ using expression (14).^27,84,85^ In this expression, SI(*λ*) is the spectral irradiance, *hc*/*λ* is the photon energy, *λ* is the wavelength of the incident light, *e* is the unit charge, *c* is the speed of light, and *K*_*J*_SC__ is a correction parameter.14
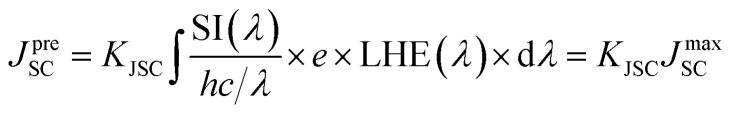


**Fig. 17:**
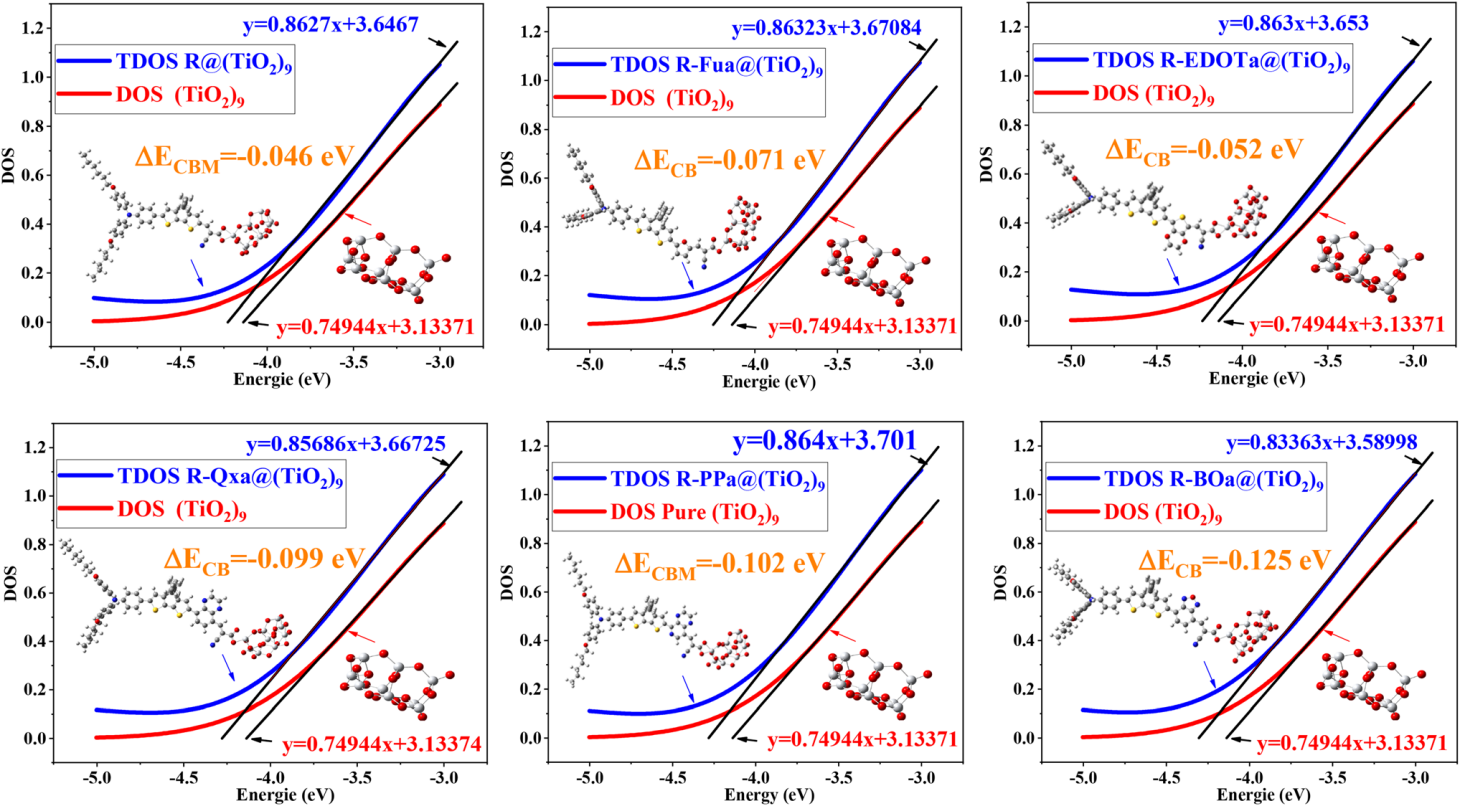
DOS of (TiO_2_)_9_ cluster and PDOS of the free organic compounds with linear fits and Δ*E*_CB_.

The correction factor *K*_*J*_SC__ can be calculated using expression (15). In this expression, *E*_CBM_ is the energy of the CB when the dye is adsorbed on the TiO_2_, which is obtained by adding *E*_C_ to Δ*E*_CB_. The value of Δ*E*_CB_ is determined as illustrated in Fig. 17 by the difference between the intercepts at the energy axis of the TDOS for dye@(TiO_2_)_9_ and the DOS for pure (TiO_2_)_9_.15
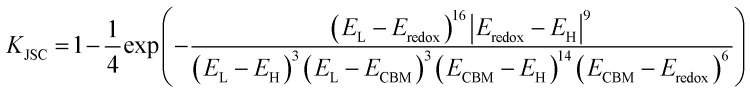


The results in Table 12 show that the *J*_SC_ of the reference dye takes 13.4 mA cm^−2^, which is in good agreement with the experimental value of 13.5 mA cm^−2^ (ref. 34) and an absolute error estimated at 0.71%. This result confirms the credibility of the proposed model for calculating and correcting the *J*_SC_. On the other hand, the *J*_SC_ follows the order R (13.4 mA cm^−2^) < R-Fua (16.73 mA cm^−2^) < R-EDOTa (17.75 mA cm^−2^) < R-Qxa (21.81 mA cm^−2^) < R-PPa (23.44 mA cm^−2^) < R-BOa (26.16 mA cm^−2^), indicating that our proposition for inserting an Ap leads to enhance the *J*_SC_ and improves the PCE. Indeed, the R-BOa will be more efficient than other dyes in the series.

**Table 12:** Predicted values of *E*_CBM_ (in eV), *K*_*J*_SC__, *n*_CB_ (in cm^−3^), *J*_SC_pre (in mA cm^−2^), *V*_OC_pre (in V), FF^pre^, and PCE (*η* in %)

Dyes	*E*_CBM_ (eV)	*K*_*J*_SC__	*J*_SC_max (mA cm^−2^)	*J*_SC_pre (mA cm^−2^)	nCB (cm^−3^)	*V*_OC_pre (V)	FF^pre^	*η* (%)
R	−4.046	0.759	17.66	13.40	1.68 × 10^20^	0.717	0.744	7.15 ± 0.15
R-Fua	−4.071	0.750	22.07	16.55	2.07 × 10^20^	0.698	0.738	8.52 ± 0.17
R-EDOTa	−4.052	0.750	23.67	17.75	2.22 × 10^20^	0.718	0.745	9.50 ± 0.19
R-Qxa	−4.099	0.750	28.77	21.58	2.70 × 10^20^	0.676	0.738	10.77 ± 0.22
R-PPa	−4.102	0.751	31.21	23.44	2.93 × 10^20^	0.676	0.733	11.62 ± 0.23
R-BOa	−4.125	0.751	35.94	26.99	3.37 × 10^20^	0.656	0.703	12.45 ± 0.25

#### 3.9.2. Predicted *V*_OC_

After the prediction of *J*_SC,_ we can estimate the *V*_OC_ by employing expression (16).^26^ In this expression, *E*_CBM_, *E*_redox_, *e*, *T*, *K*_B_, *n*_C_, and *N*_CB_ represent the conduction band energy of the adsorbed dye, redox potential of (I^−^/I_3_^−^), unit charge, absolute temperature (∼300 K), Boltzmann's constant (8.617 × 10^−5^ eV/*k*), the active density state in the CB (∼7 × 10^20^ cm^−3^), and the number of the injected electrons in CB of the semiconductor.16
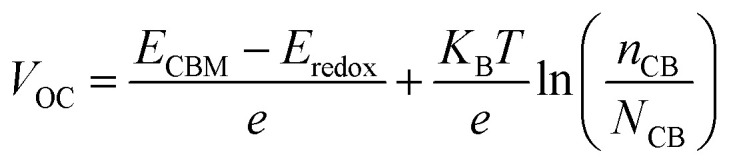


The n_CB_ parameter is predicted using expression (17).^27,84^ In this expression, *t*_b_ (∼1 s) and *d*_TiO_2__ (5 μm), which represent the time for injected electron buildup and the thickness of the semiconductor (5 μm), respectively, are introduced to make both parameters (*n*_CB_ and *N*_CB_) of the same unit.17
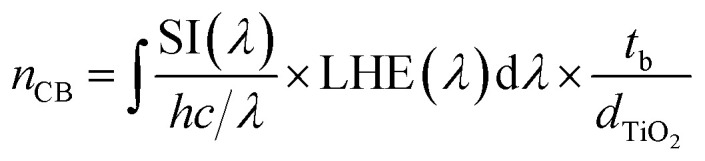


According to our prediction, the calculated *V*_OC_ of the reference takes 0.717 V, which is in excellent agreement with the experimental result of 0.721 V^34^ with an absolute error estimated at 0.57%. This result confirms the credibility of the proposed model for estimating the *V*_OC_. In addition, the predicted *V*_OC_ of the D-π-Ap-A designed dyes takes 0.718 V for R-EDOTa, 0.696 V for R-Fua, 0.676 V for R-Qxa, 0.676 V for R-PPa, and 0.656 V for R-BOa.

#### 3.9.3. Predicted *J*–*V* curves, *P*–*V* curves, and FF

It is known in the literature that the equivalent circuit of the DSSCs can be simulated as a single diode characterized by eqn (18).^86,87^18
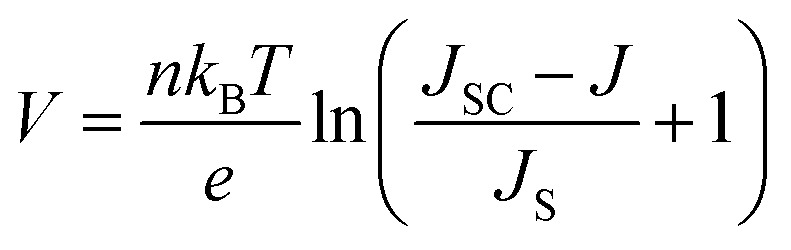
where *n* represents the diode ideality factor, *T* is the absolute temperature (300 K), *k*_B_ is Boltzmann's constant, *e* is the elementary charge, and *J*_S_ represents the saturation current density calculated using eqn (19).^86,87^19
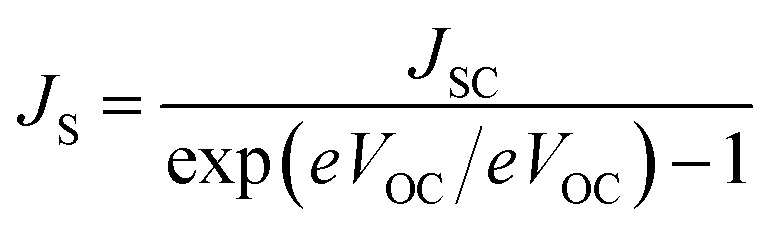


**Fig. 18:**
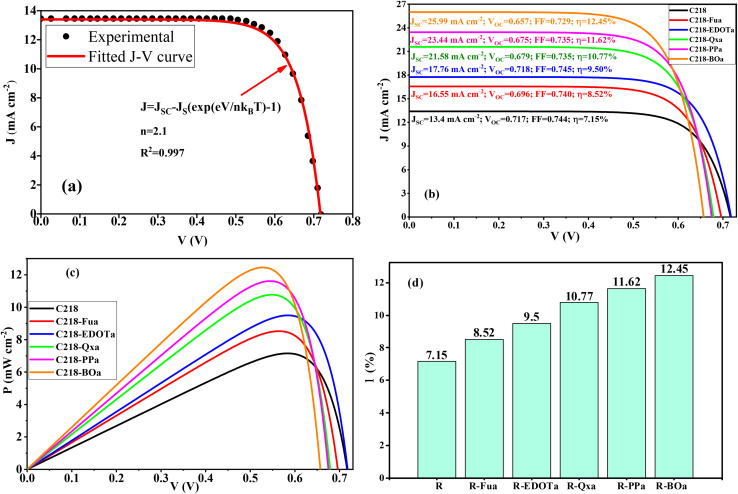
Experimental and fitted *J*–*V* curves for the reference dye (a), simulated electron transfer results (b), simulated *J*–*V* and *P*–*V* curves (c), and estimated PCE (d) for the studied dyes.

To determine the value of the ideality factor for the reference dye, we have employed the experimental *J*–*V* and fit it using the diode equation (Fig. 18). It can be seen from this figure that the fitted curve and the experimental curve^34^ are in good agreement with an ideality factor equal *n* = 2.1 and *R*^2^ = 0.99. So, to simulate the *J*–*V* and *P*–*V* curves for the designed dyes, we assume a comparable diode behaviour by applying the same ideality factor. This assumption is justified by the fact that all dyes are modelled within the same device architecture. The plotted *J*–*V* and *P*–*V* curves in Fig. 18 become broader when an Ap is inserted in the reference dye. When the *P*–*V* curves are simulated, we can determine the maximum power and estimate the fill factor FF^pre^ using eqn (20). The predicted FF values are illustrated in Table 12.20
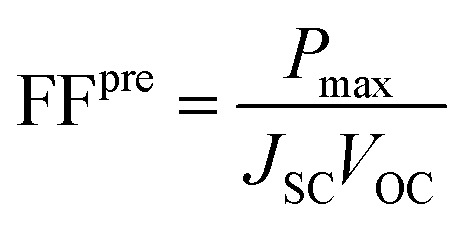


The predicted FF values are between 0.703 and 0.745. For the R dye, FF takes 0.744, which is closer to the experimental value of 0.753,^22^ with an absolute error estimated at 1.20%, confirming the credibility and validity of our estimating methodology.

#### 3.9.4. Predicted PCE (*η*)

The predicted value of the PCE (*η*) can be calculated using expression (21).21
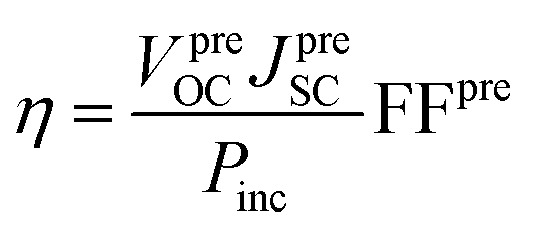
where *P*_inc_ represents the incident solar power (100 mW cm^−2^) under AM 1.5 irradiation.

The estimated values of the PCE are summarized in Table 12 and illustrated in Fig. 18(d). The predicted PCE of the reference dye takes 7.15%, this value agrees well with the experimental results of 7.3% with an absolute error estimated at 2%, confirming the credibility and validity of our estimating methodology. In addition, the maximum predicted PCE is obtained for the R-BOa dye (12.45%) followed by R-PPa (11.62%) > R-Qxa (10.77) > R-EDOTa (9.50%) > R-Fua (8.52%) > R (7.15%), indicating that all designed D-π-Ap-A organic compounds are more efficient than the reference dye. These results show that the insertion of the Fu, EDOT, Qx, PP, and BO groups as an Ap in the reference compound enhances the PCE of DSSC. In addition, the R-BOa dye is selected as the efficient dye in the series.

## 4. Conclusion

In this work, we have incorporated furan (Fu), 3,4-Ethylenedioxythiophene (EDOT), quinoxaline (Qx), pyrido(3,4-b) pyrazine (PP), and 2,1,3-benzooxadiazole (BO) as auxiliary electron acceptors (Ap) to construct five new D-π-Ap-A sensitizers based on C218 reference dye. These dyes are investigated and studied using DFT and TD-DFT methods to determine the crucial factors that can help us estimate the photovoltaic parameters. In summary, the crucial conclusion points are:

(1) The potential redox of the (*I*^−^_3_/*I*^−^) couple is positioned above the HOMOs of the studied organic compounds, and the CB of the semiconductor is positioned under their LUMOs, confirming the spontaneity of electron injection and dye regeneration.

(2) Incorporation of Fu, EDOT, Qx, PP, and BO as auxiliary electron-withdrawing groups leads to a reduction in the gap energy and consequently improves and redshifts the UV-vis absorption than the reference dye. Indeed, the emission spectra are improved and shifted to the red region than the absorption spectra.

(3) All dyes are chemisorbed spontaneously on the TiO_2_ surface. Furthermore, the adsorbed dyes show narrow gaps and broader spectra than the free dyes.

(4) The reorganization energies and the chemical reactive parameters confirm that inserting an auxiliary electron-withdrawing group Ap in D-π-A reference dye ameliorates the ICT.

(5) The hole electron investigation confirms that the electronic transition intramolecular occurs mainly by charge electron transfer from the donor group to the anchoring group.

(6) The comparison between the estimated theoretical and experimental photovoltaic parameters for the R dye show that the *J*_SC_ take 13.4 than 13.5 13.5 mA cm^−2^, *V*_OC_ take 0.717 than 0.721 V, FF take 0.744 than 0.753, and PCE take 7.15 than 7.30%, indicate a good agreement between them, confirming the validity of our estimating methodology.

(7) Compared to D-π-A reference compound, all PCEs of the designed D-π-Ap-A compound are higher and the maximum value is obtained for R-BOa (12.45%) followed by R-PPa (11.62%) > R-Qxa (10.77%) > R-EDOTa (9.50%) > R-Fua (8.52%) > R (7.15%), indicating that our methodology can provide theoretical guidance for synthesizing the new dyes that can be used as efficient sensitizers in DSSCs.

## Conflicts of interest

There are no conflicts to declare.

## Supplementary Material

RA-015-D5RA04785D-s001

## Data Availability

No specific additional data is available for this study. All cited data can be found in the manuscript and the ESI.†

## References

[cit1] Chen X., Xu B., Mei C., Ding Y., Li K. (2018). Appl. Energy.

[cit2] Prajapat K., Dhonde M., Sahu K., Bhojane P., Murty V., Shirage P. M. (2023). J. Photochem. Photobiol., C.

[cit3] Chapin D. M., Fuller C. S., Pearson G. L. (1954). J. Appl. Phys..

[cit4] Buitrago E., Novello A. M., Meyer T. (2020). Helv. Chim. Acta.

[cit5] Khir H., Pandey A., Saidur R., Ahmad M. S., Abd Rahim N., Dewika M., Samykano M. (2022). Sustain. Energy Technol. Assess..

[cit6] O'regan B., Grätzel M. (1991). Nature.

[cit7] Ding S., Yang C., Yuan J., Li H., Yuan X., Li M. (2023). RSC Adv..

[cit8] Wu J., Lan Z., Lin J., Huang M., Huang Y., Fan L., Luo G., Lin Y., Xie Y., Wei Y. (2017). Chem. Soc. Rev..

[cit9] Lee K.-M., Lin L.-C., Chen C.-Y., Suryanarayanan V., Wu C.-G. (2014). Electrochim. Acta.

[cit10] Zhao A., Huang S., Huang J., Hu P., Mao H., Chen C., Li Y., Wei M. (2021). Sol. Energy.

[cit11] Xiao Y., Han G. (2015). J. Power Sources.

[cit12] Pang Z., Zhao Y., Duan Y., Duan J., Tang Q., Yu L. (2019). J. Energy Chem..

[cit13] Venkatesan S., Chuang I.-T., Teng H., Lee Y.-L. (2023). ACS Sustain. Chem. Eng..

[cit14] Wang G., Kuang S., Zhang W. (2016). Mater. Lett..

[cit15] Bbumba S., Kigozi M., Karume I., Yiga S., Nsamba H. K., Ntale M. (2025). Dis. Nano.

[cit16] Chen M., Shao L.-L., Qian X., Liu L., Ren T.-Z., Yuan Z.-Y. (2014). Chem. Eng. J..

[cit17] Xia J., Chen L., Yanagida S. (2011). J. Mater. Chem..

[cit18] Dwivedi G., Munjal G., Bhaskarwar A. N., Chaudhary A. (2022). Inorg. Chem. Commun..

[cit19] Ramki K., Venkatesh N., Sathiyan G., Thangamuthu R., Sakthivel P. (2019). Org. Electron..

[cit20] Mahmood A. (2016). Sol. Energy.

[cit21] Sathiyan G., Sivakumar E., Ganesamoorthy R., Thangamuthu R., Sakthivel P. (2016). Tetrahedron Lett..

[cit22] Öztürk N., Bekmez M. G., Arslan B. S., Bulut E., Avcı D., Şişman İ., Nebioğlu M. (2024). Dyes Pigm..

[cit23] Kumar V., Chetti P. (2023). J. Mol. Graphics Modell..

[cit24] Saputra R. M., Yang C., Zhao D., Zheng X., Li Y. (2022). Comput. Theor. Chem..

[cit25] Zheng D., Yang X., Čuček L., Wang J., Ma T., Yin C. (2024). J. Cleaner Prod..

[cit26] Zhao C., Zhang Z., Ran X., Zhang T., Yu X., Jin L. (2024). Spectrochim. Acta, Part A.

[cit27] Yang Z., Li K., Lin C., Devereux L. R., Zhang W., Shao C., Cole J. M., Cao D. (2020). ACS Appl. Energy Mater..

[cit28] Han M.-L., Zhu Y.-Z., Liu S., Liu Q.-L., Ye D., Wang B., Zheng J.-Y. (2018). J. Power Sources.

[cit29] Mao L., Wu Y., Jiang J., Guo X., Heng P., Wang L., Zhang J. (2020). J. Phys. Chem. C.

[cit30] Rahman A. U., Khan M. B., Yaseen M., Rahman G. (2021). ACS Omega.

[cit31] Duan T., Hsiao T.-Y., Chi Y., Chen X., He Y., Zhong C. (2016). Dyes Pigm..

[cit32] Heng P., An B., Ren H., Hu Y., Guo X., Mao L., Wang L., Zhang J. (2020). J. Phys. Chem. C.

[cit33] Li R., Liu J., Cai N., Zhang M., Wang P. (2010). J. Phys. Chem. B.

[cit34] Wang P., Yang L., Wu H., Cao Y., Zhang J., Xu N., Chen S., Decoppet J.-D., Zakeeruddin S. M., Grätzel M. (2018). Joule.

[cit35] Li H., Wu Y., Geng Z., Liu J., Xu D., Zhu W. (2014). J. Mater. Chem. A.

[cit36] Wu G., Zhang Y., Kaneko R., Kojima Y., Shen Q., Islam A., Sugawa K., Otsuki J. (2017). J. Phys. Chem. C.

[cit37] Wang X., Jiang P., Chen Y., Luo H., Zhang Z., Wang H., Li X., Yu G., Li Y. (2013). Macromolecules.

[cit38] Ying W., Yang J., Wielopolski M., Moehl T., Moser J.-E., Comte P., Hua J., Zakeeruddin S. M., Tian H., Grätzel M. (2014). Chem. Sci..

[cit39] Zhang X., Mao J., Wang D., Li X., Yang J., Shen Z., Wu W., Li J., Ågren H., Hua J. (2015). ACS Appl. Mater. Interfaces.

[cit40] Hu F., Zhao Q., Li Y., Liu X. (2021). Int. J. Electrochem. Sci..

[cit41] Yashwantrao G., Saha S. (2022). Dyes Pigm..

[cit42] Chang D. W., Lee H. J., Kim J. H., Park S. Y., Park S.-M., Dai L., Baek J.-B. (2011). Org. Lett..

[cit43] Yang L.-N., Li S.-C., Li Z.-S., Li Q.-S. (2015). RSC Adv..

[cit44] Lu X., Jia X., Wang Z.-S., Zhou G. (2013). J. Mater. Chem. A.

[cit45] Sharmoukh W., Cong J., Ali B. A., Allam N. K., Kloo L. (2020). ACS Omega.

[cit46] He J., Hua J., Hu G., Yin X. J., Gong H., Li C. (2014). Dyes Pigm..

[cit47] Lee M.-W., Kim J.-Y., Lee D.-H., Ko M. J. (2014). ACS Appl. Mater. Interfaces.

[cit48] Jo H. J., Nam J. E., Heo H., Kim D.-H., Kim J., Kang J. K. (2018). J. Phys. Chem. C.

[cit49] Li J.-Y., Chen C.-Y., Ho W.-C., Chen S.-H., Wu C.-G. (2012). Org. Lett..

[cit50] Zhang G., Bala H., Cheng Y., Shi D., Lv X., Yu Q., Wang P. (2009). Chem. Commun..

[cit51] Frisch M. J. , Trucks G. W., Schlegel H. B., Scuseria G. E., Robb M. A., Cheeseman J. R., Scalmani G., Barone V., Mennucci B., Petersson G. A., et al., Gaussian 09, Revision A.1, Gaussian Inc., Wallingford CT, 2009, pp. 214–218

[cit52] Heyd J., Scuseria G. E. (2004). J. Adv. Chem. Phys..

[cit53] Chai J.-D., Head-Gordon M. (2008). Phys. Chem. Chem. Phys..

[cit54] Becke A. D. (1992). J. Adv. Chem. Phys..

[cit55] Yanai T., Tew D. P., Handy N. C. (2004). Chem. Phys. Lett..

[cit56] Adamo C., Barone V. (1998). J. Adv. Chem. Phys..

[cit57] Khadiri A., Warad I., Safi Z. S., Touhami M. E., Oudda H., Zarrouk A. (2023). J. Photochem. Photobiol., A.

[cit58] Khadiri A., Warad I., Abuelizz H. A., Touhami M. E., Oudda H., Zarrouk A. (2024). Sol. Energy.

[cit59] Liu Z., Lu T., Chen Q. (2020). Carbon.

[cit60] Lu T., Chen F. (2012). J. Comput. Chem..

[cit61] Xu Z., Li Y., Zhang W., Yuan S., Hao L., Xu T., Lu X. (2019). Spectrochim. Acta, Part A.

[cit62] Maqsood M. H., Khera R. A., Mehmood R. F., Akram S. J., Al-Zaqri N., Ibrahim M. A., Noor S., Waqas M. (2023). J. Mol. Graphics Modell..

[cit63] Al-Qurashi O. S., Wazzan N. (2021). Mol. Phys..

[cit64] Ramasamy A. K., Rajamanickam G., Bangaru S., Perumalsamy R. (2024). Comput. Theor. Chem..

[cit65] Slimi A., Hachi M., Fitri A., Benjelloun A. T., Elkhattabi S., Benzakour M., Mcharfi M., Khenfouch M., Zorkani I., Bouachrine M. (2020). J. Photochem. Photobiol., A.

[cit66] Mandal S., Kandregula G. R., Tokala V. N. B. (2020). J. Photochem. Photobiol., A.

[cit67] Britel O., Fitri A., Benjelloun A. T., Slimi A., Benzakour M., Mcharfi M. (2022). J. Photochem. Photobiol., A.

[cit68] Xu Y., Xu X., Li M., Lu W. (2020). Sol. Energy.

[cit69] Zhao D., Saputra R. M., Song P., Yang Y., Ma F., Li Y. (2020). Sol. Energy.

[cit70] Ying X., Liu Y., Ling L., Xu M., Bao B., Bao X., Pang A., Shao G., Zhang Y., Fang J. K. (2019). Sol. RRL.

[cit71] Li Y., Liu J., Liu D., Li X., Xu Y. (2019). Comput. Mater. Sci..

[cit72] Sánchez-de-Armas R., San Miguel M. A., Oviedo J., Sanz J. F. (2012). Phys. Chem. Chem. Phys..

[cit73] Pastore M., De Angelis F. (2012). Phys. Chem. Chem. Phys..

[cit74] Makowska-Janusik M., Filipecka-Szymczyk K., Pelczarski D., Stampor W., Zalas M. (2025). Molecules.

[cit75] Jaramillo-Fierro X., Capa L. F., Medina F., González S. (2021). Molecules.

[cit76] Marcus R. A. (1964). Annu. Rev. Phys. Chem..

[cit77] Hosseinzadeh E., Hadipour N. L., Parsafar G. (2017). J. Photochem. Photobiol., A.

[cit78] Zhan C.-G., Nichols J. A., Dixon D. A. (2003). J. Phys. Chem. A.

[cit79] Ahmed S., Kalita D. J. (2018). J. Adv. Chem. Phys..

[cit80] Le Bahers T., Adamo C., Ciofini I. (2011). J. Chem. Theory Comput..

[cit81] Jacquemin D., Le Bahers T., Adamo C., Ciofini I. (2012). Phys. Chem. Chem. Phys..

[cit82] Ciofini I., Le Bahers T., Adamo C., Odobel F., Jacquemin D. (2012). J. Phys. Chem. C.

[cit83] Ma W., Jiao Y., Meng S. (2014). J. Phys. Chem. C.

[cit84] Lin C., Liu Y., Wang G., Li K., Xu H., Zhang W., Shao C., Yang Z. (2020). ACS Omega.

[cit85] Xing F.-L., Zhang Z.-H., Yang C.-L., Wang M.-S., Ma X.-G. (2022). Sol. Energy.

[cit86] Duan L., Yi H., Xu C., Upama M. B., Mahmud M. A., Wang D., Shabab F. H., Uddin A. (2018). J. Photovolt..

[cit87] Shockley W., Read Jr W. (1952). Phys. Rev..

