# Supplementary material for: DFT and TD-DFT calculations to estimate the photovoltaic parameters of some metal-free dyes based on triphenylamine: the influence of inserting an auxiliary electron-withdrawing group on DSSC's performance
Source: RSC Adv. 2025 Jul 28;15(33):26807–29. doi: 10.1039/d5ra04785d (PMC12302405; doi:10.1039/d5ra04785d)
Supplement: RA-015-D5RA04785D-s001 [file RA-015-D5RA04785D-s001.pdf]

Supplementary Information (SI) for RSC Advances.
This journal is © The Royal Society of Chemistry 2025

DFT and TD-DFT calculations to estimate the photovoltaic parameters of some metal-free dyes based on triphenylamine: the influence of inserting an auxiliary electron-withdrawing group on DSSC's performance

A. Khadiri[1], Abhinay Thakur[2], I. Warad[3], H. Zarrok[1], H. Oudda[1], M. Beraich[4,5], L. Bazzi[6], A. Zarrouk[6,7,*]

[1]*Laboratory of Advanced Materials and Process Engineering, Faculty of Sciences, Ibn Tofail University, P.O. Box. 133, 14000, Kenitra, Morocco*
[2]*Division of Research and Development, Lovely Professional University, Phagwara, Punjab, 144411, India*
[3]*Department of Chemistry, AN-Najah National University, P.O. Box 7, Nablus, Palestine*
[4]*Laboratory of Applied Sciences for Sustainable Development, Higher School of Technology of El Kelaa Des Sraghna, Cadi Ayyad University, Marrakech, Morocco*
[5]*Laboratory of Materials, Energy and Environment, Faculty of Sciences Semlalia, Cadi Ayyad University, Marrakech, Morocco*
[6]*Laboratory of Industrial Engineering, Energy and Environment (LI3E), SupMTI Rabat, Morocco*
[7]*Laboratory of Materials, Nanotechnology and Environment, Faculty of Sciences, Mohammed V University in Rabat, Av. Ibn Battouta, PO Box 1014, Agdal-Rabat, Morocco*

Corresponding author
Prof. Dr. Abdelkader Zarrouk
Email: azarrouk/gmail.com (AZ)
Phone: 00212665201397
Scopus Author ID: 36125763200
https://orcid.org/0009-0003-2175-7280

Table SD1

HOMO, LUMO, and energy gap (in eV) of the reference dye (R) calculated by employing DFT in conjunction with different functionals and basis sets in CH_2Cl_2 solvant, and the relative error (pesented in % in parentheses) with experimental HOMO (-4.99 [1]).

Basis set	Energies (eV)	CAM-B3LYP	HSEH1PBE	MPW1PW91	WB97XD	B3LYP
6-31G	HOMO	-6.05 (21%)	-4.78 (4%)	-5.11 (2.4%)	*-6.64* (33%)	-4.89 (2%)
	LUMO	-1.78	-3.01	-2.67	*-1.33*	-2.88
	Gap	4.27	1.77	2.44	*5.31*	2.01
6-311G	HOMO	-6.26 (25%)	-4.95 (1%)	-5.33 (7%)	-6.80 (26%)	-5.13 (3%)
	LUMO	-1.98	-3.17	-2.99	-1.44	-3.07
	Gap	4.28	1.78	2.34	5.36	2.06
6-31G(d)	HOMO	-5.99 (20%)	-4.73 (5%)	-5.06 (1.4%)	-6.57 (32%)	-4.81 (4%)
	LUMO	-1.70	-2.96	-2.61	-1.22	-2.67
	Gap	4.29	1.77	2.45	5.35	2.14
6-311G(d)	HOMO	-6.22 (25%)	-4.92 (1.4%)	-5.25 (5%)	-6.76 (35.5%)	*-5.04 (1%)*
	LUMO	-1.93	-3.13	-2.78	-1.39	*-2.86*
	Gap	4.29	1.79	2.47	5.37	*2.18*
6-31G(d,p)	HOMO	-6.00 (20%)	-4.74 (5%)	-5.06 (1.4%)	-6.58 (32%)	-4.81 (4%)
	LUMO	-1.71	-2.96	-2.61	-1.23	-2.68
	Gap	4.29	1.78	2.45	5.35	2.13
6-311G(d,p)	HOMO	-6.22 (25%)	-4.91 (2%)	-5.29 (6%)	-6.76 (35.5%)	***-5.04*** (1%)
	LUMO	-1.93	-3.13	-2.84	-1.40	*-2.87*
	Gap	4.29	1.77	2.45	5.36	*2.17*

Table SD2

The maximum absorption wavelength λ_{max} of the reference dye (R) calculated by employing TD-DFT in conjunction with different functionals and 6-311G (d, p) basis set under CPCM in CH_2Cl_2 solvant.

HSEH1PBE	B3LYP	MPW1PW91	WB97XD	CAM-B3LYP	Exp
663	650	596	474	***512***	471 [2], 517 [3], 520 [4], 546 [5], 549 [1], 555 [6], 556 [7]

Tables

Table SD3

Excited state parameters of the free dyes carried out by employing TD-DFT/CAM-B3LYP/6-311G** under CPCM in CH_2Cl_2 solvent.

Dyes	State	λ_{max} (nm)	E_{ex} (eV)	f	Main compositions (%)
R	S_1	512 (520[a], 549[b], 555[c])	2.42	1.83	H → L (65); H-1 → L (29)
	S_2	362	3.42	0.15	H-1 → L (43); H → L+1 (19); H → L (17); H-3 → L (10)
	S_3	310	4.00	0.18	H → L+1 (50); H → L (13); H-1 → L (10)
	S_5	287	4.32	0.12	H-7 → L (21); H-3 → L (14); H-4 → L (13); H-1 → L+1 (10)
	S_6	276	4.49	0.40	H-7 → L+2 (32); H-1 → L+1 (14); H-3 → L (12); H → L+2 (12)
	S_7	273	4.54	0.35	H → L+4 (70); H-1 → L+4 (14)
	S_{10}	266	4.66	0.14	H → L+6 (63); H-1 → L+6 (15)
R-Fua	S_1	543	2.28	2.24	H → L (61); H-1 → L (27)
	S_2	387	3.20	0.11	H → L+1 (47); H-1 → L (23); H-2 → L (15)
	S_4	314	3.96	0.52	H → L+2 (30); H-2 → L (22); H-1 → L+1 (19)
	S_7	274	4.52	0.43	H → L+4 (60); H-1 → L+4 (27)
	S_{10}	267	4.64	0.12	H → L+5 (50); H-1 → L+5 (27)
R-EDOTa	S_1	557	2.23	2.23	H → L (67); H-1 → L (24)
	S_2	387	3.20	0.12	H → L+1 (43); H-1 → L (25); H-2 → L (18)
	S_3	342	3.63	0.18	H-4 → L (74)
	S_5	317	3.91	0.42	H-2 → L (24); H-1 → L+1 (17); H → L+2 (17); H-5 → L (13)
	S_8	275	4.5	0.43	H → L+4 (55); H-1 → L+4 (32)
R-Qxa	S_1	608	2.04	1.95	H → L (67); H-1 → L (24)
	S_2	400	3.10	0.28	H-1 → L (35); H → L+2 (30); H-2 → L (16)
	S_3	362	3.43	0.25	H → L+2 (42); H → L (21); H-1 → L (18)
	S_5	334	3.71	0.33	H → L+1 (41); H-1 → L+1 (26)
	S_6	320	3.87	0.16	H-2 → L (29); H-1 → L+2 (16); H-4 → L (12)
	S_9	296	4.19	0.12	H-1 → L+2 (23); H → L+6 (22); H-2 → L (15); H-1 → L (11)
	S_{10}	392	4.25	0.12	H-5 → L (26); H-6 → L (24); H-11 → L (12)
R-PPa	S_1	637	1.95	2.01	H → L (68); H-1 → L (24)
	S_2	407	3.04	0.18	H-1 → L (46); H → L+2 (18); H-3 → L (15); H → L (14)
	S_3	362	3.43	0.25	H → L+2 (57); H → L (15); H-1 → L+2 (11); H-1 → L (10)
	S_5	343	3.62	0.34	H → L+1 (48); H-1 → L+1 (29)
	S_6	321	3.86	0.14	H-3 → L (36); H-4 → L (13); H-5 → L (10)
R-BOa	S_1	710	1.75	1.99	H → L (67); H-1 → L (26)
	S_2	437	2.84	0.13	H-1 → L (47); H → L (20); H-2 → L (17)
	S_3	375	3.31	0.30	H → L+1 (35); H → L+2 (28); H → L (10)
	S_4	344	3.60	0.27	H-2 → L (41); H-4 → L (20); H → L+2 (12)
	S_5	333	3.73	0.12	H → L+1 (28); H → L+2 (21); H-1 → L+2 (21)
	S_8	304	4.08	0.17	H → L+4 (32); H-1 → L+1 (24); H-1 → L+2 (17)

Table SD4

Optical parameters of the excited states for dyes@$(TiO_2)_9$ systems in dichloromethane solvent.

Dyes	State	λ_{max} (nm)	E_{ex}	f	Main compositions (%)
R/$(TiO_2)_9$	S_1	556	2.19	2.22	H → L+1 (58); H-1 → L+1 (21)
	S_2	382	3.25	0.10	H-1 → L+1 (44); H → L+1 (15); H-3 → L+1 (10)
R-Fua/$(TiO_2)_9$	S_1	583	2.13	2.39	H → L+1 (46); H-1 → L+1 (25); H → L (11)

	S_2	399	3.11	0.12	H → L+20 (21); H-1 → L+20 (20); H-2 → L+1 (14)
R-EDOTa/$(TiO_2)_9$	S_1	593	2.09	2.41	H → L (32); H → L+1 (27); H-1 → L (15); H-1 → L+1 (13)
	S_4	357	3.47	0.18	H-4 → L (47); H-4 → L+1 (41)
	S_{10}	325	3.81	0.47	H-2 → L (15); H-2 → L+1 (10)
R-Qxa/$(TiO_2)_9$	S_1	647	1.92	2.19	H → L (67); H-1 → L (23)
	S_2	411	3.02	0.18	H-1 → L (41); H-2 → L (17); H → L+22 (16); H → L (12)
	S_4	367	3.37	0.23	H → L+22 (39); H → L+12 (14); H → L (12); H → L (11)
	S_7	347	3.57	0.10	H-10 → L (45); H-10 → L+22 (10)
	S_8	346	3.59	0.28	H-10 → L (20); H → L+22 (13)
R-PPa/$(TiO_2)_9$	S_1	672	1.84	2.28	H → L (68); H-1 → L (25)
	S_2	422	2.94	0.14	H-2 → L (48); H-1 → L (18); H-3 → L (16); H-1 → L+20 (11)
	S_4	366	3.39	0.11	H-1 → L+1 (50); H-1 → L+20 (25)
	S_6	353	3.51	0.47	H-1 → L+20 (22); H-1 → L+16 (15); H-2 → L+16 (12)
R-BOa/$(TiO_2)_9$	S_1	769	1.61	2.31	H → L (69); H-1 → L (25)
	S_2	458	2.70	0.10	H-1 → L (50); H → L (20); H-2 → L (17)
	S_3	382	3.25	0.17	H → L+16 (27); H → L+19 (24)
	S_5	354	3.50	0.44	H-2 → L (28); H → L+19 (23); H-4 → L (15)

Table SD5

Contribution of holes and electrons, the overlap and the difference between hole and electron for different groups of investigated free dyes in DCM solvent (all values in %).

Dyes	Groups	Hole	Electron	Overlap	Difference
R	D	25.16	9.71	15.63	-15.45
	Π	58.74	58.99	58.86	0.25
	A	16.10	31.30	22.45	15.20
R-Fua	D	12.02	4.90	7.67	-7.12
	Π	53.83	38.71	45.65	-15.12
	Aa	21.34	26.00	23.55	4.36
	A	12.82	30.39	19.74	17.58
R-EDOTa	D	10.75	4.83	7.20	-5.93
	Π	53.88	36.61	44.41	-17.27
	Aa	23.59	32.66	27.76	9.07
	A	11.78	25.91	17.47	14.12
R-Qxa	D	14.20	4.29	7.81	-9.91
	Π	58.38	32.97	43.87	-25.41
	Aa	19.91	43.16	29.32	23.24
	A	7.50	19.58	12.12	12.08
R-PPa	D	16.20	4.78	8.80	-11.43
	Π	57.95	36.28	45.85	-21.67
	Aa	18.98	43.43	28.71	24.45
	A	6.87	15.52	10.33	8.64
R-BOa	D	13.57	3.76	7.14	-9.81
	Π	54.55	31.21	41.26	-23.34
	Aa	22.86	46.94	32.76	24.09
	A	9.02	18.09	12.77	9.07

Equations

$$\Delta\rho(r) = \rho^{ele}(r) - \rho^{hole}(r) \quad \text{(SD1)}$$

$$D = \sqrt{D_x{}^2 + D_y{}^2 + D_z{}^2} \quad \text{(SD2)}$$

$$H_{CT} = \left(|\delta_{ele}| + |\delta_{hole}|\right)/2 \quad \text{(SD3)}$$

$$t = D - H_{CT} \quad \text{(SD4)}$$

$$S_r = \int \sqrt{\rho^{hole}(r)\rho^{ele}(r)}dr \quad \text{(SD5)}$$

$$E_C = \iint \frac{\rho^{hole}(r_1)\rho^{ele}(r_2)}{|r_1 - r_2|} dr_1 dr_2 \quad \text{(SD6)}$$

In these equations, ρ^{hole} and ρ^{ele} represent the density distribution of hole and electron, respectively, D is the distance between hole and electron centroids, D_x, D_y and D_z are the distances between centroids of hole and electron in corresponding directions, H_{CT} is the half of the sum of two centroid axis along the electron transfer direction, δ_{ele} and δ_{hole} present the spatial distance distribution of electrons and holes, t measure separation degree of hole and electron in CT direction, S_r is the overlap function between hole and electron distribution, and E_C is the Coulomb attractive energy between hole and electron.

Figures

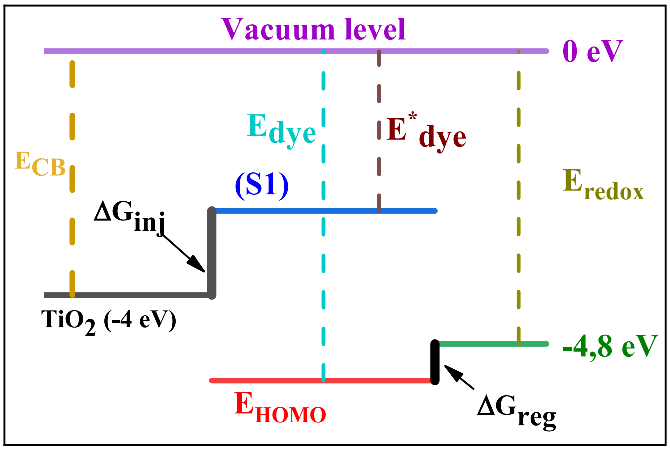

Fig.SD1. Representation of driving force of electron injection and dye regeneration with energy arrangement of DSSC.

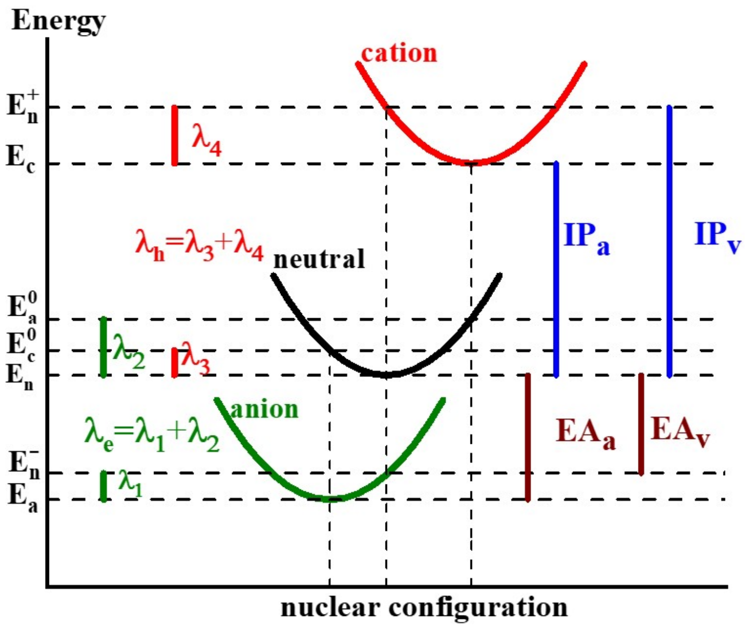

Figure SD2. Representation of potential energy curves for the neutral, anionic, and cationic molecules.

References

1. A. Yella, R. Humphry-Baker, B. F. Curchod, N. Ashari Astani, J. Teuscher, L. E. Polander, S. Mathew, J.-E. Moser, I. Tavernelli and U. Rothlisberger, *Chemistry of Materials*, 2013, **25**, 2733-2739.
2. A. Dualeh, F. De Angelis, S. Fantacci, T. Moehl, C. Yi, F. Kessler, E. Baranoff, M. K. Nazeeruddin and M. Grätzel, *The Journal of Physical Chemistry C*, 2012, **116**, 1572-1578.
3. M. Xu, D. Zhou, N. Cai, J. Liu, R. Li and P. Wang, *Energy & Environmental Science*, 2011, **4**, 4735-4742.
4. J. Liu, J. Zhang, M. Xu, D. Zhou, X. Jing and P. Wang, *Energy & Environmental Science*, 2011, **4**, 3021-3029.
5. Y. Tan, M. Liang, Z. Lu, Y. Zheng, X. Tong, Z. Sun and S. Xue, *Organic Letters*, 2014, **16**, 3978-3981.
6. R. Li, J. Liu, N. Cai, M. Zhang and P. Wang, *The Journal of Physical Chemistry B*, 2010, **114**, 4461-4464.
7. W. H. Nguyen, C. D. Bailie, J. Burschka, T. Moehl, M. Grätzel, M. D. McGehee and A. Sellinger, *Chemistry of Materials*, 2013, **25**, 1519-1525.
